# Comprehensive Bayesian analysis of rare (semi)leptonic and radiative $\varvec{B}$ decays

**DOI:** 10.1140/epjc/s10052-014-2897-0

**Published:** 2014-06-03

**Authors:** Frederik Beaujean, Christoph Bobeth, Danny van Dyk

**Affiliations:** 1 C2PAP, Universe Cluster, Ludwig-Maximilians-Universität München, Garching, Germany; 2 Universe Cluster and Institute for Advanced Study, Technische Universität München, Garching, Germany; 3 Theoretische Elementarteilchenphysik, Universität Siegen, Siegen, Germany

## Abstract

The available data on $|\Delta B| = |\Delta S| = 1$ decays are in good agreement with the Standard Model when permitting subleading power corrections of about $15\,\%$ at large hadronic recoil. Constraining new-physics effects in $\mathcal {C}_{7}^{\mathrm {}}$, $\mathcal {C}_{9}^{\mathrm {}}$, $\mathcal {C}_{10}^{\mathrm {}}$, the data still demand the same size of power corrections as in the Standard Model. In the presence of chirality-flipped operators, all but one of the power corrections reduce substantially. The Bayes factors are in favor of the Standard Model. Using new lattice inputs for $B\rightarrow K^*$ form factors and under our minimal prior assumption for the power corrections, the favor shifts toward models with chirality-flipped operators. We use the data to further constrain the hadronic form factors in $B\rightarrow K$ and $B\rightarrow K^*$ transitions.

## Introduction

The rare $B$ decays mediated by $b\rightarrow s \gamma$ and $b\rightarrow s \ell ^+\ell ^-$ ($\ell = e,\, \mu ,\, \tau$) flavor-changing neutral-current transitions are important probes of the Standard Model (SM) and provide constraints on nonstandard effects in the flavor sector up to the TeV range. In recent years, phenomenological analyses focused on the exclusive modes $B\rightarrow K^{*}(\rightarrow K \pi )\, \ell ^+\ell ^-$, $B\rightarrow K \ell ^+\ell ^-$, and $B_s \rightarrow \mu ^+\mu ^-$. Many observables of these modes were recently measured at the LHC (LHCb, CMS, and ATLAS), and previously at $B$ factories (Belle, BaBar) and the Tevatron (CDF) [1–19].

The goal is to measure a large number of observables accessible in the angular analysis of the decay distributions of the three- and four-body final states. With the CP-averaged observables one can test—in a model-independent fashion—the underlying short-distance couplings of the $\Delta B = 1$ effective theory. CP-asymmetric observables also probe new sources of CP violation beyond the SM. In $B\rightarrow K^{*}(\rightarrow K \pi )\, \ell ^+\ell ^-$ decays, certain combinations of the angular observables, the “optimized observables” [20–28], are free of form factors to leading order in the $1/m_b$ expansion and consequently expected to have smaller theoretical uncertainties. The same framework also provides observables that are dominated by ratios of $B\rightarrow K^*$ form factors [22, 28–31], providing some additional data-driven control over these hadronic quantities.

The $1/m_b$ expansions are important tools for the prediction of exclusive decays. At large hadronic recoil of the $K^{(*)}$ meson, QCD factorization (QCDF) yields corrections beyond naive factorization [32, 33]. Effects of ($q\bar{q}$)-resonances, dominantly from charm, as well as the chromomagnetic dipole operator can be calculated using a light-cone operator product expansion (OPE) in combination with dispersion relations [34–38]. At low hadronic recoil, a local OPE [39, 40] can be employed. Its prediction of correlations between different observables can be tested experimentally through measurement of the observables $H_T^{(1)}$ and $J_7$ in $B\rightarrow K^*\ell ^+\ell ^-$ [28]. Hadronic form factors are a major source of theoretical uncertainties in the prediction of angular observables, whereas optimized observables are sensitive to higher-order terms in the $1/m_b$ expansions, especially at large recoil. At present, the associated uncertainties due to the unknown $1/m_b$ contributions are estimated based on simple power-counting arguments.

As of 2011, several global analyses of the available data—differing in the degree of sophistication, the statistical approach, and the estimation of theory uncertainties—have been performed [27, 30, 41–44]. Recently, experimental updates from LHCb [5, 15, 16, 45, 46] and CDF [14] became available as well as analogous measurements from CMS [6, 17] and ATLAS [18]. LHCb is the first experiment to measure optimized observables [19]. Perhaps the most outstanding LHCb result is that the measured value of $P_{4,5}'$ is in some tension with the SM predictions, stimulating new global fits [47–49].

Based on the framework developed in our previous work [30], we perform a global analysis using Bayesian inference, and we include a total of 28 nuisance parameters to account for theory uncertainties. Apart from new-physics parameters, our framework allows us to infer also $B\rightarrow K^{(*)}$ form factors and the size of subleading contributions, thereby shedding some light on the origin of tensions between data and SM predictions. Compared to [30], we include the most recent measurements, add additional observables to the fit, and account also for recent lattice calculations of form factors [50, 51] and other new theoretical results.

In Sect. 2, the model-independent framework of $\Delta B = 1$ decays is briefly revisited, and three scenarios of new physics (NP) are introduced. In Sect. 3, we list the updated experimental input. The results of the global analysis are presented in Sect. 4: (1) for the most important Wilson coefficients of the SM operator basis $\mathcal {C}_{7,9,10}^{\mathrm {}}$ and their chirality-flipped counterparts assuming them to be real-valued, (2) for the $B\rightarrow K^{(*)}$ form factors in the SM and the two NP scenarios, and (3) the size of subleading contributions. We also compare our results with recent analyses that had access to the same experimental data. In Appendix A we summarize the theoretical predictions of newly included observables and changes in the treatment of form factors and subleading contributions.

## Model-independent scenarios 

For the global analysis of $b\rightarrow s (\gamma ,\, \ell ^+\ell ^-)$ data we use a model-independent approach based on the $|\Delta B| = |\Delta S| = 1$ effective theory. The Hamiltonian reads

2.1 $$\begin{aligned} \mathcal{{H}}_\mathrm{eff}= - \frac{4\, G_F}{\sqrt{2}} V_{tb}^{} V_{ts}^*\,\frac{\alpha _e}{4 \pi }\, \sum _i \mathcal {C}_{i}^{\mathrm {}}(\mu ) \mathcal{O}_i + \text {h.c.} \end{aligned}$$

with dimension-six flavor-changing operators $\mathcal{O}_i$ and their respective short-distance couplings, the Wilson coefficients $\mathcal {C}_{i}^{\mathrm {}}(\mu )$. We evaluate the hadronic matrix elements of the operators at the scale $\mu = 4.2 \, \text{ GeV }$ of the order of the bottom-quark mass $m_b$. We restrict our analysis to the set of operators present in the SM ($i = 7, 9, 10$)

2.2 $$\begin{aligned} \begin{array}{ll} &{}\!\!\!\mathcal{O}_{7(7')} = \frac{m_b}{e}\!\big [\bar{s} \sigma ^{\mu \nu } P_{R(L)} b\big ] F_{\mu \nu }, \\ &{}\!\!\!\mathcal{O}_{9(9')} = \big [\bar{s} \gamma _\mu P_{L(R)} b\big ]\,\big [\bar{\ell } \gamma ^\mu \ell \big ], \\ &{}\!\!\!\mathcal{O}_{10(10')} = \big [\bar{s} \gamma _\mu P_{L(R)} b\big ]\,\big [\bar{\ell } \gamma ^\mu \gamma _5 \ell \big ] \end{array} \end{aligned}$$

and their chirality-flipped counterparts ($i = 7',9',10'$), denoted by SM$'$. The Wilson coefficients of the four-quark and the chromomagnetic dipole operators are set to their NNLO SM values at $\mu = 4.2 \,\text{ GeV }$ [52, 53].

In principle, the scalar, pseudo-scalar, and tensor $b\rightarrow s \ell ^+\ell ^-$ operators can contribute to the angular distributions of $B\rightarrow K^*(\rightarrow K \pi ) \ell ^+\ell ^-$ and $B\rightarrow K \ell ^+\ell ^-$ as discussed in great detail in [28]. However, only a few measurements of sensitive observables are currently available, with rather large uncertainties. We therefore abstain from including these operators into our analysis. Instead, we focus on comparing the SM to several new-physics scenarios with regard to their ability to describe the data well.

In the model-independent approach, the SM and SM$'$ Wilson coefficients are the parameters of interest; they are assumed real valued and independent *a priori*. The nuisance parameters $\varvec{\nu }$ serve to model theory uncertainties, including CKM parameters, quark masses, and hadronic matrix elements; see Appendix A. The following scenarios of fit parameters:

2.3 $$\begin{aligned}&\text {SM(}\nu {-only)} : {\left\{ \begin{array}{ll} \mathcal {C}_{7,9,10}^{\mathrm {}} &{} \text {SM values}\\ \mathcal {C}_{7',9',10'}^{\mathrm {}} &{} \text {SM values}\\ \varvec{\nu }&{} \text {free floating} \end{array}\right. },\nonumber \\&\text {SM} : {\left\{ \begin{array}{ll} \mathcal {C}_{7}^{\mathrm {}} &{} \in [-2,+2]\\ \mathcal {C}_{9,10}^{\mathrm {}} &{} \in [-15,+15]\\ \mathcal {C}_{7',9',10'}^{\mathrm {}} &{} \text {SM values}\\ \varvec{\nu }&{} \text {free floating} \end{array}\right. }, \end{aligned}$$

$$\begin{aligned}&\text {SM+SM'{}} : {\left\{ \begin{array}{ll} \mathcal {C}_{7,7'}^{\mathrm {}} &{} \in [-1,+1]\\ \mathcal {C}_{9,9',10,10'}^{\mathrm {}} &{} \in [-7.5,+7.5]\\ \varvec{\nu }&{} \text {free floating} \end{array}\right. },\\&\text {SM+SM'(9){}} : {\left\{ \begin{array}{ll} \mathcal {C}_{7,7',10,10'}^{\mathrm {}} &{} \text {SM values}\\ \mathcal {C}_{9,9'}^{\mathrm {}} &{} \in [-7.5,+7.5]\\ \varvec{\nu }&{} \text {free floating} \end{array}\right. }. \end{aligned}$$

Expressing vague prior knowledge, we assign a flat prior distribution to the Wilson coefficients. However, each nuisance parameter $\varvec{\nu }$ comes with an informative prior as discussed in detail in Appendix A.

The SM values $\mathcal {C}_{7,9,10}^{\mathrm {}}$ are obtained at NNLO [52, 53] and depend on the fundamental parameters of the top-quark and the $W$-boson masses, as well as on the sine of the weak mixing angle. For new-physics models that fall into one of the scenarios SM and SM+SM$'$, the obtained fit results of the Wilson coefficients can be subsequently used to constrain those models’ fundamental parameters after accounting for the renormalization group evolution from the high matching scale down to $\mu \sim m_b$.

## Observables and experimental input 

In this section we describe changes of the experimental inputs that enter our global analysis with respect to our previous work [30]. We first introduce observables which are newly added to the global analysis and refer the reader for details of their theoretical treatment to Appendix A. Afterward, we summarize those observables whose measurements have been updated, or for which additional measurements have since become available. In general we employ the full set of observables listed in Table 1 except for the last row and denote this as “full”. Inclusion of the $B\rightarrow K^*$ lattice points from the last row is denoted as “full (+FF)”. For the sake of comparison with [47] we also repeat the analysis with a smaller subset called “selection” as specified in the same table. Generally, we model the probability distributions of experimental measurements as (multivariate) Gaussian distributions. In practice, however, experiments do not yet provide correlations, except for $S$ and $C$ in $B\rightarrow K^*\gamma$. For measurements with asymmetric uncertainties, we model the probability distribution as a split Gaussian with two different widths. For the measurement of $\mathcal{B}(B_s \rightarrow \mu ^+\mu ^-)$, we use the Amoroso distribution to avoid the unphysical region $\mathcal{B}< 0$ as described in [30].

**Table 1:** List of all observables in the various inclusive and exclusive $b\rightarrow s (\gamma ,\, \ell ^+\ell ^-)$ decays that enter the global fits with their respective kinematics and experiments that provide the measurements. The $B^*$–$B^{}$ mass splitting is used to constrain matrix elements of dimension-five operators. Lattice results of $B\rightarrow K$ form factors are used to constrain their parameters, and theoretical constraints on $B\rightarrow K^*$ form factors are included. For more details we refer to Sect. 3 and Appendix A . $\dagger$: Note that we include only the LHCb measurements in the $[1,\,6]$ GeV$^2$ bin as part of the “selection” data set, but not the low-recoil bins

Channel	Constraints	Kinematics	Source	Selection
$B\rightarrow X_s \gamma$	$\mathcal{B}$	$1.8\,\text{ GeV } < E_\gamma$	[1, 2]	$\checkmark$
$B\rightarrow X_s \ell ^+\ell ^-$	$\mathcal{B}$	$q^2\in [1,\, 6]$ GeV$^2$	[3, 4]	$\checkmark$
$B_s\rightarrow \mu ^+\mu ^-$	$\int \mathrm{d}\tau \mathcal{B}(\tau )$	–	[5, 6]	$\checkmark$
$B\rightarrow K^*\gamma$	$\mathcal{B}$, $S$, $C$	–	[7–11]	$\checkmark$
${B\rightarrow K \ell ^+\ell ^-}$	$\mathcal{B}$	$q^2\in [1,\, 6],\, [14.18,\, 16],\, [> 16]$ GeV$^2$	[12–14]	–
		$q^2 \in [1,\, 6],\, [14.18,\, 16],\, [16,\, 18],\, [18, \, 22]$ GeV$^2$	[15]	–
${B\rightarrow K^* \ell ^+\ell ^-}$	$\mathcal{B}$	$q^2\in [1,\,6],\, [14.18,\, 16],\, [> 16]$ GeV$^2$	[12–14, 16, 17]	–
	$F_L$	$-$"$-$	[12–14, 16–18]	–
	$A_\mathrm{FB}$	$-$"$-$	[12–14, 16–18]	$\dagger$
	$A_T^{(2)}$	$-$"$-$	[14, 16]	$\dagger$
	$A_T^\mathrm{re}$, $P'_{4,5,6}$	$-$"$-$	[16, 19]	$\dagger$
$B$ properties	$M_{B^*} - M_{B^{}}$	–	[54]	$\checkmark$
${B\rightarrow K}$ form factor	$f_+$	$q^2 = 17,\, 20,\, 23$ GeV$^2$	[50]	–
${B\rightarrow K^*}$ form factors	$V/A_1$	$q^2 = 0$ GeV$^2$	Large energy limit	$\checkmark$
	$A_0$	$q^2 = 0$ GeV$^2$	[35]	$\checkmark$
	$V$, $A_1$, $A_{12}$	$q^2 = 15,\, 19.21$ GeV$^2$	[51]	–

In the following, all observables are understood to be CP-averaged unless noted otherwise. The dilepton invariant mass in inclusive and exclusive $b\rightarrow s\ell ^+\ell ^-$ decays is denoted by $q^2$ throughout.

### New observables

Measurements of the branching ratio of the inclusive radiative decay $B\rightarrow X_s \gamma$

3.1 $$\begin{aligned} \mathcal{B}_{1.8\,\mathrm{GeV}}&= (3.36 \pm 0.13 \pm 0.25) \cdot 10^{-4},&[1]\end{aligned}$$

3.2 $$\begin{aligned} \mathcal{B}_{1.8\,\mathrm{GeV}}&= (3.21 \pm 0.15 \pm 0.29) \cdot 10^{-4},&[2] \end{aligned}$$

are included with a lower cut on the photon energy $E_\gamma > 1.8$ GeV. For the branching ratio of the inclusive semileptonic decay $B\rightarrow X_s \ell ^+\ell ^-$ integrated over the low-$q^2$ region $q^2\in [1,\, 6]$ GeV$^2$, we use

3.3 $$\begin{aligned} \langle \mathcal{B}\rangle _{[1, 6]}&= (1.8 \pm 0.7 \pm 0.5) \cdot 10^{-6},&[3]\end{aligned}$$

3.4 $$\begin{aligned} \langle \mathcal{B}\rangle _{[1, 6]}&= (1.493 \pm 0.504 {}^{+0.411}_{-0.321}) \cdot 10^{-6}.&[4] \end{aligned}$$

A source of parametric uncertainty in inclusive decays arises from matrix elements of dimension-five operators, $\mu ^2_{\pi }$ and $\mu ^2_{G}$, discussed in more detail in Appendix A.1. They appear in the heavy-quark expansion at order $\Lambda _\mathrm{QCD}^2/m_b^2$, and $\mu ^2_{G}$ enters also the $B^*$–$B$ mass splitting [54]

3.5 $$\begin{aligned} M_{B^*} - M_{B^{}}&= (4.578 \pm 0.035) \cdot 10^{-2}\,\text{ GeV } , \end{aligned}$$

incorporated as an additional experimental constraint.

Previous experimental angular analyses of $B\rightarrow K^{*} (\rightarrow K \pi )\, \ell ^+ \ell ^-$ were restricted to the measurements of the longitudinal $K^*$-polarization fraction, $F_L$, and the lepton forward-backward asymmetry, $A_\mathrm{FB}$. The CDF collaboration was the first to measure $A_T^{(2)}$ and the CP-asymmetry $A_9 = A_{im}$ [55]. Most recently, LHCb extended the angular analysis to measure $A_T^\mathrm{re}$ [16] as well as $S_{4,5,7,8}$ and their optimized analogs $P_{4,5,6,8}'$ [19] in addition to the previously published results on $A_T^{(2)}$, $S_3$, $A_9$, and $S_9$. The original definitions of the observables $S_i$ and $P_i'$ can be found in [56] and [27], respectively. Here we include the measurements of $A_T^\mathrm{re}$ and $P_{4,5,6}'$ in the $q^2$-bins $[1,\,6]$, $[14.18,\,16.0]$, and $[>\!\!16.0]$ GeV$^2$. We replace $S_3$ data from LHCb by the corresponding $A_T^{(2)}$ results.

### Updated experimental input

We use the same experimental input as in our previous analysis [30], unless the experimental collaborations provide updated measurements. For some of the observables, additional measurement by further experimental collaborations have become available; they are added to the previous ones. Both types of updates are listed below.

The measurement of the time-integrated and CP-averaged branching ratio of the leptonic decay $B_s \rightarrow \mu ^+\mu ^-$ has been recently updated by LHCb and measured for the first time by CMS

3.6 $$\begin{aligned} \mathcal{B}&= \left( 2.9 {}^{+1.1}_{-1.0} {}^{+0.3}_{-0.1}\right) \cdot 10^{-9},&[5]\end{aligned}$$

3.7 $$\begin{aligned} \mathcal{B}&= \left( 3.0 {}^{+1.0}_{-0.9}\right) \cdot 10^{-9},&[6] \end{aligned}$$

with $4.0\,\sigma$ and $4.3\,\sigma$ signal significance, respectively. To faithfully model the physical constraint $\mathcal{B}\ge 0$ and the reported asymmetric uncertainties of the experimental probability distribution (PDF), we use the Amoroso distribution [30].

The LHCb measurement of the $B^+ \rightarrow K^+ \ell ^+\ell ^-$ branching ratio (CP-averaged) [15], based on $1$ fb$^{-1}$ integrated luminosity, is used in the $q^2$ bins $[1,\,6]$, $[14.18, \, 16.0]$, $[16.0, \, 18.0]$, and $[18.0,\,22.0]$ GeV$^2$, in addition to the previous results from Belle and BaBar. The new CDF results are now based on the full [14] rather than a partial data set [57]. Very recently, LHCb reported a broad peaking structure in the branching ratio at high $q^2$ compatible with $\psi (4160)$ [58] using the larger data set of $3$ fb$^{-1}$. Within the picture of quark-hadron duality, an adapted larger bin $[\ge \!15]$ GeV$^2$ around the peak should satisfy the necessary conditions required by the theoretical framework [39, 40] in the future. For the moment, we continue to use the binning provided by experiments and do not enlarge theoretical uncertainties in $B \rightarrow K^{(*)} \ell ^+\ell ^-$ at high $q^2$.

There are numerous updates on $B\rightarrow K^* \ell ^+ \ell ^-$ for which we use the three $q^2$ bins $[1,\,6]$, $[14.18,\,16.0]$, and $[>\!16.0]$ GeV$^2$. We add recent measurements of the branching ratio from CMS [17] as well as for $F_L$ and $A_\mathrm{FB}$ from CMS [17] and ATLAS [18]. For the branching ratio, $F_L$, and $A_\mathrm{FB}$ we now use the updated values [16] instead of [59] in the case of LHCb, and [14] instead of [55, 57] in the case of CDF.

For $B^0\rightarrow K^{*0}(\rightarrow K \pi )\, \ell ^+\ell ^-$ LHCb provides results of “optimized observables”. Combining these with both the observables $F_L$ and $A_\mathrm{FB}$ can lead to double counting [25] in the strict limit of vanishing lepton masses that is well justified in the NP scenarios considered in this work.^1^ For this reason, we replace the LHCb measurements of $\langle A_\mathrm{FB}\rangle _{[1,6]}$ by $\langle A_T^\mathrm{re}\rangle _{[1,6]}$. However, we continue to use $\langle A_\mathrm{FB}\rangle$ rather than $\langle A_T^\mathrm{re}\rangle$ for the low-recoil bins because neither is an optimized observable at high $q^2$. It is difficult to include the LHCb measurement of $\langle A_T^\mathrm{re} \rangle _{[14.18, 16.0]}$, since it implies that $A_T^\mathrm{re}(q^2)$ is constant and attains its theoretical maximum.

Overall, the measured $q^2$ dependence of individual observables is in quite good agreement with the SM predictions. The largest deviation of $3.7\sigma$ is reported for the optimized observable $\langle P_5' \rangle _{[4.3,8.68]}$ [19] when compared to the SM prediction [47]. We do not use any of the $[4.3,8.68]$ GeV$^2$ bins since their theory predictions receive large contributions from $c\bar{c}$-loops [35]. The $[1,6]$ GeV$^2$ bins are less affected by these effects. The remaining uncertainty is accounted for by the parameters of subleading contributions at the level of the decay amplitudes [30] in our predictions. The SM predictions available in the literature for this bin (and the low-recoil bins) are compared with our results in Table 2. Based on our prior input, we obtain central values as in [47] deviating by $2.5\sigma$ from the measurement whereas the analysis [38] has a different central value and larger errors with a $1.0\sigma$ deviation from experiment.

**Table 2:** Predictions based on SM($\nu$-only) and wide (tripled uncertainty) priors, and postdictions after the fits, for the optimized observable $P'_{4,5,6}$ in various $q^2$ bins; see Appendix A.3 for details. We compare our results with several sources. Note that for our predictions (postdictions) the uncertainties correspond to $68\,\%$ credibility intervals that arise from variation of only the nuisance parameters (all fit parameters). $\dagger$: Values have been adjusted to match the theory convention for the observable

Source	$\langle P'_4 \rangle _{[1,6]}$	$\langle P'_4 \rangle _{[14.18,16]}$	$\langle P'_4 \rangle _{[16,19]}$	$\langle P'_5\rangle _{[1,6]}$	$\langle P'_5 \rangle _{[14.18,16]}$	$\langle P'_5 \rangle _{[16,19]}$	$\langle P'_6\rangle _{[1,6]}$	$\langle P'_6\rangle _{[14.18,16]}$	$\langle P'_6 \rangle _{[16,19]}$
Measurement									
LHCb$^\dagger$ [19]	$0.58^{+0.32}_{-0.36}$	$-0.18^{+0.54}_{-0.70}$	$0.70^{+0.44}_{-0.52}$	$+0.21^{+0.20}_{-0.21}$	$-0.79^{+0.27}_{-0.22}$	$-0.60^{+0.21}_{-0.18}$	$+0.18^{+0.21}_{-0.21}$	$0.18^{+0.24}_{-0.25}$	$-0.31^{+0.38}_{-0.39}$
Predictions in SM($\nu$-only)									
Nominal priors	$0.47^{+0.07}_{-0.08}$	$+1.21^{+0.08}_{-0.10}$	$1.30^{+0.05}_{-0.05}$	$-0.34^{+0.09}_{-0.08}$	$-0.77^{+0.16}_{-0.14}$	$-0.56^{+0.13}_{-0.13}$	$-0.07^{+0.01}_{-0.02}$	$\mathcal {O}\left( {10^{-4}}\right)$	$\mathcal {O}\left( {10^{-4}}\right)$
Wide priors	$0.44^{+0.15}_{-0.15}$	$+1.21^{+0.08}_{-0.10}$	$1.31^{+0.04}_{-0.07}$	$-0.32^{+0.18}_{-0.10}$	$-0.77^{+0.16}_{-0.14}$	$-0.54^{+0.13}_{-0.17}$	$-0.07^{+0.02}_{-0.03}$	$\mathcal {O}\left( {10^{-4}}\right)$	$\mathcal {O}\left( {10^{-4}}\right)$
Ref. [38]	$0.46^{+0.16}_{-0.19}$	–	–	$-0.28^{+0.30}_{-0.26}$	–	–	$-0.07^{+0.08}_{-0.10}$	–	–
Ref. [47]	$0.56^{+0.07}_{-0.06}$	$+1.16^{+0.19}_{-0.33}$	$1.26^{+0.12}_{-0.25}$	$-0.35^{+0.09}_{-0.10}$	$-0.78^{+0.33}_{-0.36}$	$-0.60^{+0.28}_{-0.37}$	$-0.09^{+0.04}_{-0.05}$	$0.00^{+0.00}_{-0.00}$	$0.00^{+0.00}_{-0.00}$
Ref. [49]	–	$+1.22^{+0.38}_{-0.38}$	$1.30^{+0.02}_{-0.02}$	–	$-0.71^{+0.07}_{-0.07}$	$-0.54^{+0.04}_{-0.04}$	–	–	–
Postdictions: this work, “full” data set									
SM($\nu$-only)	$0.49^{+0.06}_{-0.04}$	$+1.13^{+0.03}_{-0.03}$	$1.24^{+0.02}_{-0.02}$	$-0.23^{+0.02}_{-0.03}$	$-0.84^{+0.05}_{-0.04}$	$-0.65^{+0.04}_{-0.04}$	$-0.08^{+0.01}_{-0.01}$	$\mathcal {O}\left( {10^{-4}}\right)$	$\mathcal {O}\left( {10^{-4}}\right)$
SM	$0.51^{+0.07}_{-0.08}$	$+1.12^{+0.03}_{-0.03}$	$1.24^{+0.02}_{-0.03}$	$-0.24^{+0.03}_{-0.03}$	$-0.85^{+0.04}_{-0.05}$	$-0.66^{+0.04}_{-0.05}$	$-0.08^{+0.01}_{-0.01}$	$\mathcal {O}\left( {10^{-4}}\right)$	$\mathcal {O}\left( {10^{-4}}\right)$
SM+SM$'$	$0.51^{+0.05}_{-0.06}$	$+1.22^{+0.03}_{-0.05}$	$1.30^{+0.02}_{-0.03}$	$-0.26^{+0.04}_{-0.03}$	$-0.71^{+0.05}_{-0.08}$	$-0.55^{+0.06}_{-0.05}$	$-0.08^{+0.01}_{-0.01}$	$\mathcal {O}\left( {10^{-4}}\right)$	$\mathcal {O}\left( {10^{-4}}\right)$

A second interesting deviation appears in the optimized observable $\langle P_4' \rangle _{[14.18,16.0]}$. In the SM operator basis it is given by a ratio of form factors [31] up to strongly suppressed subleading corrections. The extrapolation of LCSR form-factor results [35] from low to high $q^2$ yields a much larger value compared to the measurement. This is also observed [49] with recent lattice QCD determinations of $B\rightarrow K^*$ form factors at high $q^2$ [51]. They allow us to further constrain the form factor parameters *a priori*. However, these determinations do not reliably consider systematic uncertainties due to finite width effects [51] of the $K^*$. For this reason, we provide our fit results for analyses with and without the $B\rightarrow K^*$ lattice inputs. Note, however, that this issue does not affect the lattice determinations of $B\rightarrow K$ form factors.

A third deviation from the SM prediction is seen in the preliminary measurements of $\langle F_L \rangle _{[1,6]}$ from BaBar and ATLAS that are both too low by more than $3\sigma$ and $2\sigma$, respectively. This stands in contrast to the published results of Belle, CDF, CMS, and LHCb, which are all in good agreement with the SM at low $q^2$. The BaBar results are an average of $B^0 \rightarrow K^{*0} \ell ^+\ell ^-$ and $B^+ \rightarrow K^{*+} \ell ^+\ell ^-$. While the neutral mode yields $F_L$ consistent or close to the SM, the charged mode deviates strongly in the low $q^2$ region and points in principle to a large isospin asymmetry for the longitudinally polarized $K^*$ branching fraction [60]. The ATLAS measurement has been performed only for the neutral mode. Although preliminary and despite the isospin average in the case of BaBar, we include both measurements in the fit.

## Results 

In this section, we review briefly our statistical approach and summarize our fit results in several subsections, providing measures for the goodness of fit. We describe several solutions in the subspace of the Wilson coefficients for all four scenarios introduced in Sect. 2. Using Bayes factors, we compare models with non-SM Wilson coefficients to the SM($\nu$-only) fit. Finally, we present results for the nuisance parameters of the form factors and subleading corrections to the $B\rightarrow K^*\ell ^+\ell ^-$ transition amplitudes in each of the scenarios. Throughout we will compare with recent similar analyses in the literature.

### Statistical approach

Our results are obtained from a Bayesian fit, similar to our previous work [30]. The main outputs are samples drawn from the posterior distribution using the EOS flavor program [61]. The samples are obtained using an algorithm that employs Markov chains, hierarchical clustering, and adaptive importance sampling (for a detailed description we refer to [62]).

Throughout we denote by $P(\varvec{\theta }\,| D,M)$ the posterior, where $D\in \lbrace$full, full (+FF), selection$\rbrace$ represents the data set, and $\varvec{\theta }$ all parameters ($\mathcal {C}_{i}^{\mathrm {}}$ and nuisance parameters $\varvec{\nu }$) of the model $M \in \lbrace$SM($\nu$-only), SM, SM+SM$'$, SM+SM$'(9)$ $\rbrace$ as defined in Sect. 2. The weighted posterior samples provide access to all marginal distributions and to the evidence

4.1 $$\begin{aligned} P(D|M) = \int _{V_0} \text{ d } \, \varvec{\theta } \,\, P(D|\varvec{\theta },M)\, P_0(\varvec{\theta }\, | M ), \end{aligned}$$

where the integration extends over the whole prior volume $V_0$ spanned by the parameters $\varvec{\theta }$. The likelihood and prior distribution are denoted by $P(D|\varvec{\theta },M)$ and $P_0(\varvec{\theta }\,|M)$, respectively. For $M\in \lbrace$SM, SM+SM$'$ $\rbrace$, the posterior has numerous well separated local maxima. Most of these have a negligible impact, and we consider only those solutions with significant posterior mass. We require the ratio $R$ of the local evidence—integration volume $V_0$ restricted to contain only a single solution—to the global evidence (4.1) exceeds $0.001$. We label the individual solutions as $A$ in the SM($\nu$-only) scenario, $A$ and $B$ in the SM scenario, and $A'$ through $D'$ in the SM+SM$'$ scenario, whereas in SM+SM$'(9)$ only $A'$ appears. For each model, $A^{(\prime )}$ denotes the solution in which the signature of $(\mathcal {C}_{7}^{\mathrm {}}, \mathcal {C}_{9}^{\mathrm {}}, \mathcal {C}_{10}^{\mathrm {}})$ is $(-,+,-)$ as predicted in the SM($\nu$-only), and $B^{(\prime )}$ indicates flipped signs; i.e., $(+,-,+)$.

To determine the goodness of fit, we first find the best-fit point, $\varvec{\theta }^{*}$, in each solution using Minuit [63]. Next, we calculate the pull value as in [30] for fixed $M$, $\varvec{\theta }^{*}$ for each constraint, and finally $\chi ^2$ as the quadratic sum of pulls. From $\chi ^2$, a $p$ value follows assuming $N_\mathrm{dof}$ degrees of freedom. Note that there are $N$ experimental constraints and $\dim \varvec{\nu }$ informative priors. For the goodness of fit, we consider each informative prior as *one* constraint. With $K$ Wilson coefficients varied in $M$, we have

4.2 $$\begin{aligned} N_\mathrm{dof} = (N + \dim \varvec{\nu }) - (K + \dim \varvec{\nu }) = N - K \end{aligned}$$

degrees of freedom. For the full data set we have $\dim \varvec{\nu }= 28$ nuisance parameters, and we include $N=93$ constraints. In addition there are 11 or 5 theory constraints on the form factors, depending on whether we include the lattice results of the $B\rightarrow K^*$ form factors or not. Below we denote both setups as “full (+FF)” and “full”, respectively. For the selection data set, we have $N=27$ inputs—including two theory constraints—and $\dim \varvec{\nu }= 23$. For this data set we do not include the lattice form factor results since their theory uncertainty, when extrapolated to low $q^2$, is comparable to the uncertainty of the LCSR results.

We calculate the Bayes factor between two statistical models $M_1$ and $M_2$ and for a common data set $D$,

4.3 $$\begin{aligned} B(D|M_1,M_2 ) \equiv \frac{P(D|M_1)}{P(D|M_2)}. \end{aligned}$$

The standard quantity to compare two models, the posterior odds, are defined as

4.4 $$\begin{aligned} \frac{P(M_1 | D)}{P(M_2|D)} = B(D|M_1, M_2) \frac{P_0(M_1)}{P_0(M_2)}; \end{aligned}$$

i.e., the product of the Bayes factor and the prior odds $P_0(M_1) / P_0(M_2)$. It is important to note that the prior of the model parameters, $P_0(\varvec{\theta }| M)$, is an integral part of the model $M$. Therefore, the Bayes factor penalizes $M_2$ versus $M_1$ if $M_2$ contains extra parameters because the evidence (4.1) is just the likelihood weighted by the prior, and the average typically decreases when the same unit probability mass is smeared over a larger volume $V_0$. This occurs, e.g., in the present analysis for $M_1=$ SM($\nu$-only) and $M_2=$ SM as the Wilson coefficients are fixed in $M_1$ but variable with flat priors over a large volume covering multiple solutions in $M_2$. With the evidence given separately for each solution in Table 3, the reader can, for example, compute the Bayes factor between $M_1$ and $M_2$ as though only one of the solutions had been allowed a priori by reducing the (flat) prior ranges of the Wilson coefficients and scaling the evidence accordingly. We focus on the SM-like solution of each scenario that is fully contained in a hyperrectangle with edge lengths

4.5 $$\begin{aligned} 0.4&\quad \text{ for }&\mathcal {C}_{7,7'}^{\mathrm {}},\nonumber \\ 4.0&\quad \text{ for }&\mathcal {C}_{9,9', 10, 10'}^{\mathrm {}}. \end{aligned}$$

The penalty due to extra parameters can be overcome if $M_2$ provides a significantly better description of the data; i.e., higher likelihood values. In conclusion, $B(D|M_1, M_2) > 1$ implies that the data favor $M_1$.

We stress that the evidence by itself is not meaningful because of the arbitrary likelihood normalization due to the fact that we do not have the actual events seen by an experiment but only a concise summary usually in the form of an observable’s value maximizing the likelihood value plus uncertainties. Using a consistent normalization, at least ratios of evidences for identical data $D$—as in the Bayes factor—have a well defined interpretation.

**Table 3:** Goodness of fit and posterior evidence (ratio) for various combinations of constraints and fit models. Individual solutions are labeled as $A$ and $B$ in the SM($\nu$-only) and the SM, and $A'$ through $D'$ in the SM+SM$'$ and $A'$ in the SM+SM$'(9)$. The solutions with SM-like and flipped signs of $\mathcal {C}_{i}^{\mathrm {}}$ are $A^{(\prime )}$ and $B^{(\prime )}$, respectively. For the definitions of $P(D|M)$ and $R$ see (4.1) and below

Scenario	Data set	Solution	$\chi ^2$	$p$ value	$\ln P(D|M)$	$R$
SM($\nu$-only)	Full	$A$	111.1	0.10	571.9	1
	Full (+FF)	$A$	118.4	0.04	576.6	1
	Selection	$A$	19.9	0.75	110.9	1
SM	Full	$A$	109.6	0.08	561.0	0.74
		$B$	110.0	0.08	560.0	0.26
	Full (+FF)	$A$	118.1	0.03	565.7	0.70
		$B$	118.6	0.02	564.9	0.30
	Selection	$A$	14.6	0.88	103.3	0.47
		$B$	14.3	0.89	103.5	0.53
SM+SM$'$	Full	$A'$	104.8	0.09	560.4	0.39
		$B'$	105.3	0.09	560.5	0.41
		$C'$	106.8	0.07	558.3	0.05
		$D'$	106.3	0.08	559.5	0.15
	Full (+FF)	$A'$	105.0	0.09	569.6	0.49
		$B'$	105.5	0.09	569.2	0.31
		$C'$	107.8	0.06	567.4	0.05
		$D'$	106.9	0.07	568.4	0.15
SM+SM$'(9)$	Full	$A'$	106.6	0.13	571.3	1
	Full (+FF)	$A'$	107.4	0.12	580.7	1

The fit results regarding model comparison and goodness of fit are listed in Table 3, in which the local evidence is available at a relative precision of 1 % on the linear scale. For an overview of all of the following results on the Wilson coefficients, we refer to Table 4. The pull values entering the $p$ value are compiled for each experimental constraint in Table 5 for the solution $A$ of SM and $A'$ of the SM+SM$'$.

**Table 4:** The 68- and 95 %-credibility intervals and the local modes of the marginalized 1D posterior distributions of the Wilson coefficients at $\mu = 4.2$ GeV, $P(\mathcal {C}_{i}^{\mathrm {}}|D),\, i = 7,9,10,7',9',10'$, for nominal priors of nuisance parameters in the various scenarios. Note that for the SM+SM$'$ scenario the individual solutions cannot be disentangled within the 1D posterior distributions, unlike for the SM scenario. For comparison, the SM values of the Wilson coefficients read $\mathcal {C}_{7}^{\mathrm {SM}} = -0.34$, $\mathcal {C}_{9}^{\mathrm {SM}} = +4.27$, $\mathcal {C}_{10}^{\mathrm {SM}} = -4.17$, $\mathcal {C}_{7'}^{\mathrm {SM}} = -0.01$, $\mathcal {C}_{9'}^{\mathrm {SM}} = \mathcal {C}_{10'}^{\mathrm {SM}} = 0$

Scenario		SM “selection”		SM “full”		SM+SM$'$ “full”			
Solution		$A$	$B$	$A$	$B$				
$\mathcal {C}_{7}^{\mathrm {}}$	$68\,\%$	[$-0.37,\,-0.32$]	[$+0.48,\,+0.54$]	[$-0.38,\,-0.31$]	[$+0.50,\,+0.51$]	[$-0.38,\,-0.31$] $\cup$ [$+0.47,\,+0.53$]			
	$95\,\%$	[$-0.39,\,-0.30$]	[$+0.46,\,+0.56$]	[$-0.46,\,-0.28$]	[$+0.46,\,+0.55$]	[$-0.40,\,-0.29$] $\cup$ [$-0.02,\,+0.20$] $\cup$ [$+0.45,\,+0.55$]			
	Mode	$-0.34$	$+0.51$	$-0.34$	$+0.51$	$-0.33$ $\wedge$ $+0.12$ $\wedge$ $+0.50$			
$\mathcal {C}_{9}^{\mathrm {}}$	$68\,\%$	[$+2.46,\,+3.54$]	[$-4.70,\,-3.45$]	[$+3.49,\,+4.58$]	[$-5.13,\,-4.96$]	[$-5.05,\,-4.09$] $\cup$ [$+3.00,\,+3.68$]			
	$95\,\%$	[$+1.98,\,+4.14$]	[$-5.39,\,-2.92$]	[$+3.28,\,+4.89$]	[$-5.75,\,-4.46$]	[$-5.59,\,-3.27$] $\cup$ [$+2.73,\,+4.09$]			
	Mode	$+2.94$	$-4.02$	$+3.98$	$-5.03$	$-4.43$ $\wedge$ $+3.48$			
$\mathcal {C}_{10}^{\mathrm {}}$	$68\,\%$	[$-4.55,\,-3.71$]	[$+3.73,\,+4.67$]	[$-4.98,\,-4.12$]	[$+4.41,\,+4.55$]	[$-4.77,\,-3.68$] $\cup$ [$+2.05,\,+3.68$]			
	$95\,\%$	[$-5.01,\,-3.25$]	[$+3.25,\,+5.08$]	[$-5.18,\,-3.86$]	[$+3.90,\,+4.96$]	[$-5.05,\,-3.00$] $\cup$ [$-2.05,\,-1.64$] $\cup$ [$+2.05,\,+3.68$]			
	Mode	$-4.12$	$+4.19$	$-4.58$	$+4.48$	$-4.30$ $\wedge$ $-1.70$ $\wedge$ $+3.20$			
$\mathcal {C}_{7'}^{\mathrm {}}$	$68\,\%$	–	–	–	–	[$-0.09,\,+0.07$] $\cup$ [$+0.40,\,+0.44$]			
	$95\,\%$	–	–	–	–	[$-0.45,\,-0.42$] $\cup$ [$-0.16,\,+0.13$] $\cup$ [$+0.38,\,+0.45$]			
	Mode	–	–	–	–	$-0.43$ $\wedge$ $+0.01$ $\wedge$ $+0.43$			
$\mathcal {C}_{9'}^{\mathrm {}}$	$68\,\%$	–	–	–	–	[$-3.82,\,-1.77$] $\cup$ [$+0.82,\,+2.32$]			
	$95\,\%$	–	–	–	–	[$-4.09,\,-1.23$] $\cup$ [$-0.54,\,+3.55$]			
	Mode	–	–	–	–	$-2.39$ $\wedge$ $+2.11$			
$\mathcal {C}_{10'}^{\mathrm {}}$	$68\,\%$	–	–	–	–	[$-1.91,\,+0.40$]			
	$95\,\%$	–	–	–	–	[$-3.00,\,+1.22$] $\cup$ [$+2.18,\,+2.45$]			
	Mode	–	–	–	–	$-0.75$ $\wedge$ $+2.25$			

### Fitting the nuisance parameters

Let us begin the summary of our results with the fit of the scenario SM($\nu$-only); i.e., the fit of nuisance parameters by fixing Wilson coefficients to their values in the SM at the scale $\mu = 4.2$ GeV

4.6 $$\begin{aligned} \mathcal {C}_{7}^{\mathrm {SM}}&= -0.34,&\mathcal {C}_{9}^{\mathrm {SM}}&= 4.27,&\mathcal {C}_{10}^{\mathrm {SM}}&= -4.17. \end{aligned}$$

The main purpose is to check whether the SM($\nu$-only), including all theory uncertainties, provides a good description of the available data. This scenario serves as the reference point to compare with scenarios SM, SM+SM$'(9)$ and SM+SM$'$ later on.

Within the SM($\nu$-only) scenario, we perform three fits to the data sets “full”, “full (+FF)”, and “selection”. All fits exhibit good or satisfactory $p$ values of $0.10$, $0.04$, and $0.75$, respectively, and they show that the posterior is unimodal. For the values of the evidence and $\chi ^2$, we refer to Table 3. The large $p$ value for the data set “selection” can be explained through the smaller overall number of measurements, and especially the absence of measurements deviating substantially from their SM predictions, such as ATLAS’ and BaBar’s results on $\langle F_L\rangle _{[1,6]}$. The measurements with the largest pull values above $2\sigma$ are all in $B\rightarrow K^*\ell ^+\ell ^-$ as can be seen in Table 5: the aforementioned $\langle F_L \rangle _{[1,6]}$ from BaBar and ATLAS, $\langle \mathcal{B}\rangle _{[16,19]}$ from Belle, $\langle A_\mathrm{FB} \rangle _{[16,19]}$ from ATLAS and the two optimized observables $\langle P_4' \rangle _{[14.18,16]}$ and $\langle P_5' \rangle _{[1,6]}$ from LHCb (see also the caption of Table 5).

**Table 5:** Compilation of the pull values in units of $\sigma$ at the SM-like best fit points $A$ in the SM fit (left columns) and $A'$ in the SM+SM$'$ fit (right columns), listed per experiment and observable. Only pull values for fits with the “full” data set are listed. The single CLEO measurement of $\mathcal{B}(B\rightarrow K^*\gamma )$ has a pull value $+0.3\sigma$ in both the SM and the SM+SM$'$ fits. The pull values for the SM($\nu$-only) fit are very similar to the SM fit for the “full” data set, with the exception of $+2.1\sigma$ for the LHCb measurement of $\langle P'_5\rangle _{[1,6]}$. The pull values for SM+SM$'$ with the “full (+FF)” data set deviate by less than $0.2$ from those given for SM+SM$'$ “full”. The pull values for SM “full (+FF)” also agree with those of SM “full” except for notable deviations of $\sim 0.6\sigma$ for the high $q^2$ bins of the $B\rightarrow K^*\ell ^+\ell ^-$ branching ratio

Observable		SM full, solution $A$						SM+SM$'$ full, solution $A'$					
		ATLAS	BaBar	Belle	CDF	CMS	LHCb	ATLAS	BaBar	Belle	CDF	CMS	LHCb
$B\rightarrow X_s\gamma$	$\mathcal{B}$	–	$-0.1$	$+0.4$	–	–	–	–	$-0.1$	$+0.4$	–	–	–
$B\rightarrow X_s\ell ^+\ell ^-$	$\langle \mathcal{B}\rangle _{[1,6]}$	–	$+0.4$	$+0.0$	–	–	–	–	$+0.6$	$+0.3$	–	–	–
$B_s\rightarrow \mu ^+\mu ^-$	$\mathcal{B}$	–	–	–	–	$-1.0$	$-1.0$	–	–	–	–	$+0.5$	$+0.4$
$B\rightarrow K^*\gamma$	$\mathcal{B}$	–	$+0.7$	$-1.2$	–	–	–	–	$+0.7$	$-1.2$	–	–	–
	$S + C$	–	$+0.4$	$+0.7$	–	–	–	–	$+0.9$	$+0.4$	–	–	–
$B\rightarrow K\ell ^+\ell ^-$	$\langle \mathcal{B}\rangle _{[1,6]}$	–	$+0.0$	$+0.0$	$+0.0$	–	$-1.3$	–	$+0.2$	$+0.3$	$+0.2$	–	$-0.9$
	$\langle \mathcal{B}\rangle _{[14.18,16]}$	–	$+1.1$	$+0.4$	$+1.0$	–	$+1.0$	–	$+1.0$	$+0.2$	$+0.7$	–	$+0.4$
	$\langle \mathcal{B}\rangle _{[16,18]}$	–	–	–	–	–	$+1.2$	–	–	–	–	–	$+0.7$
	$\langle \mathcal{B}\rangle _{[16,23]}$	–	$+0.2$	$+1.7$	$-1.3$	–	–	–	$+0.0$	$+1.6$	$-1.6$	–	–
	$\langle \mathcal{B}\rangle _{[18,22]}$	–	–	–	–	–	$-0.1$	–	–	–	–	–	$-0.5$
$B\rightarrow K^*\ell ^+\ell ^-$	$\langle \mathcal{B}\rangle _{[1,6]}$	–	$+0.6$	$-0.6$	$+0.4$	$+0.9$	$-0.3$	–	$+0.5$	$-0.7$	$+0.3$	$+0.9$	$-0.4$
	$\langle \mathcal{B}\rangle _{[14.18,16]}$	–	$+1.0$	$-0.2$	$+1.0$	$-1.3$	$-0.7$	–	$+1.0$	$-0.3$	$+1.0$	$-1.3$	$-0.8$
	$\langle \mathcal{B}\rangle _{[16,19]}$	–	$-0.6$	$\varvec{+2.6}$	$-1.4$	$+0.9$	$-0.3$	–	$-0.6$	$\varvec{+2.5}$	$-1.5$	$+0.8$	$-0.3$
	$\langle A_\mathrm{FB}\rangle _{[1,6]}$	$-0.9$	$-1.9$	$-1.3$	$-1.9$	$-0.5$	–	$-1.0$	$-1.9$	$-1.3$	$-1.9$	$-0.5$	–
	$\langle A_\mathrm{FB}\rangle _{[14.18,16]}$	$-0.2$	$+0.9$	$-1.1$	$-0.5$	$+1.4$	$-1.4$	$-0.3$	$+0.8$	$-1.1$	$-0.5$	$+1.4$	$-1.5$
	$\langle A_\mathrm{FB}\rangle _{[16,19]}$	$\varvec{+2.2}$	$+0.2$	$-1.7$	$-0.1$	$-0.4$	$+1.1$	$\varvec{+2.2}$	$+0.2$	$-1.7$	$-0.1$	$-0.3$	$+1.1$
	$\langle F_L\rangle _{[1,6]}$	$\varvec{-2.6}$	$\varvec{-3.5}$	$+0.4$	$+1.2$	$+0.9$	$+0.9$	$\varvec{-2.5}$	$\varvec{-3.3}$	$+0.5$	$+1.3$	$+1.2$	$+1.2$
	$\langle F_L\rangle _{[14.18,16]}$	$-0.5$	$+0.5$	$-1.8$	$+0.7$	$+1.4$	$-0.3$	$-0.3$	$+0.6$	$-1.7$	$+1.0$	$+1.6$	$+0.0$
	$\langle F_L\rangle _{[16,19]}$	$+0.2$	$+1.2$	$-1.4$	$-1.5$	$+1.4$	$+0.6$	$+0.4$	$+1.3$	$-1.3$	$-1.4$	$+1.6$	$+0.8$
	$\langle A_T^{(2)}\rangle _{[1,6]}$	–	–	–	$-0.2$	–	$+0.5$	–	–	–	$-0.4$	–	$-0.7$
	$\langle A_T^{(2)}\rangle _{[14.18,16]}$	–	–	–	$+0.5$	–	$+1.1$	–	–	–	$+0.4$	–	$+0.7$
	$\langle A_T^{(2)}\rangle _{[16,19]}$	–	–	–	$-0.1$	–	$-0.5$	–	–	–	$-0.3$	–	$-0.7$
	$\langle A_T^{(\mathrm{re})}\rangle _{[1,6]}$	–	–	–	–	–	$+1.0$	–	–	–	–	–	$+1.0$
	$\langle P'_4\rangle _{[1,6]}$	–	–	–	–	–	$+0.1$	–	–	–	–	–	$+0.4$
	$\langle P'_4\rangle _{[14.18,16]}$	–	–	–	–	–	$\varvec{-2.4}$	–	–	–	–	–	$\varvec{-2.3}$
	$\langle P'_4\rangle _{[16,19]}$	–	–	–	–	–	$-1.2$	–	–	–	–	–	$-1.2$
	$\langle P'_5\rangle _{[1,6]}$	–	–	–	–	–	$+1.6$	–	–	–	–	–	$+1.1$
	$\langle P'_5\rangle _{[14.18,16]}$	–	–	–	–	–	$+0.1$	–	–	–	–	–	$+0.1$
	$\langle P'_5\rangle _{[16,19]}$	–	–	–	–	–	$+0.2$	–	–	–	–	–	$+0.3$
	$\langle P'_6\rangle _{[1,6]}$	–	–	–	–	–	$+1.2$	–	–	–	–	–	$+1.2$
	$\langle P'_6\rangle _{[14.18,16]}$	–	–	–	–	–	$+0.7$	–	–	–	–	–	$+0.7$
	$\langle P'_6\rangle _{[16,19]}$	–	–	–	–	–	$-0.8$	–	–	–	–	–	$-0.8$

We note that removing the ATLAS and BaBar measurements of $\langle F_L\rangle _{[1,6]}$ increases the $p$ value substantially, from $0.10$ to $0.38$ and from $0.04$ to $0.30$ for the “full” and “full (+FF)” data sets, respectively. The smaller $p$ value of the “full (+FF)” data set compared to the one of the “full” data set arises dominantly from a tension of the $\mathcal{B}(B\rightarrow K^*\ell ^+\ell ^-)$ data at high $q^2$ with predictions based on $B\rightarrow K^*$ lattice form factors [49, 51].

**Fig. 1:**
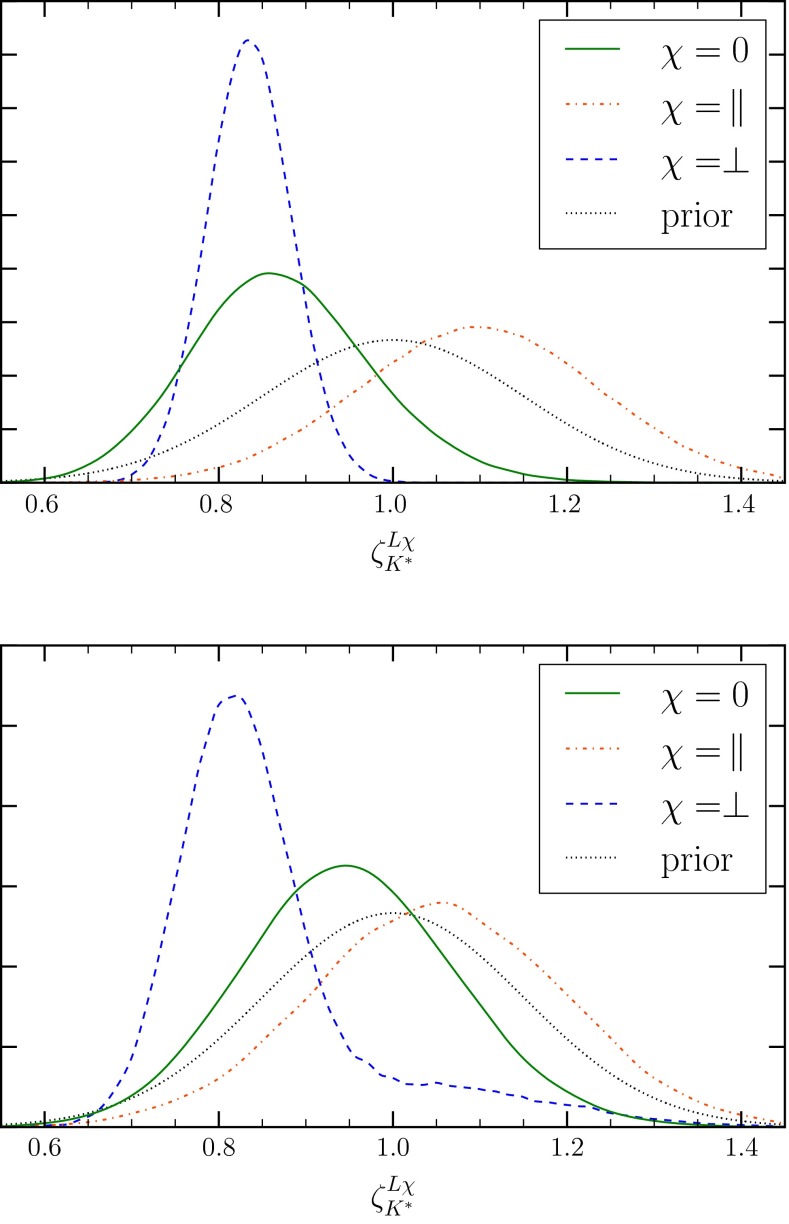
Comparison of the prior (*gray*, *dotted*) and marginalized posterior distributions, using the “full” data set, for the parameters $\zeta ^{L\chi }_{K^*}$, $\chi =\,\perp ,\parallel ,0$, describing the unknown $1/m_b$ contributions to the $B\rightarrow K^*\ell ^+\ell ^-$ transversity amplitudes $A_\chi ^{L}$ at large recoil. *Upper panel* SM($\nu$-only); in the SM, the results are very similar (not shown). *Lower panel* SM+SM$'$. Note that the tail for $\chi =\,\perp$ is a consequence of the suppressed solutions $C'$ and $D'$

We find that the impact of experimental measurements published after our previous analysis [30] does not change the main outcome; i.e., the data can be accurately described without resort to new physics beyond the SM. This result may appear surprising given the large tensions that were seen in [47]. Within our approach, however, the tension between SM prediction and measurement of $\langle P'_5\rangle _{[1,6]}$ can be eased by shifts in the parameters $\zeta ^{L \chi }_{K^*}$, $\chi = \perp ,\parallel ,0$ that parametrize the size of subleading contributions at large recoil in $B\rightarrow K^*\ell ^+\ell ^-$; see Appendix A.3 for their definition. The shifts of about $-(15$–$20)\,\%$ to $\zeta ^{L \chi }_{K^*}$, $\chi =\perp ,0$ and about $+10\,\%$ to $\zeta ^{L \parallel }_{K^*}$ are compatible with the power-counting expectation $\Lambda _\text {QCD}/m_b$. They suffice to increase the most likely value of $\langle P'_5\rangle _{[1,6]}$ from $-0.34$ (nominal prior) to $-0.23$ (see Table 2), thereby reducing the tension and explaining the $B\rightarrow K^*\ell ^+\ell ^-$ “anomaly”. The prior and posterior distributions of $\zeta ^{L \chi }_{K^*}$ are shown in Fig. 1. We do not find any shifts in the parameters $\zeta ^{R \chi }_{K^*}$, $\chi = \perp ,\parallel ,0$ exceeding a few percent. There are no significant differences between the “full” and “full (+FF)” fits, except for two shifts in subleading parameters. At low $q^2$, $\zeta ^{L 0}_{K^*}$ reduces from $-15\,\%$ to about $-5\,\%$, and at high $q^2$, $\Lambda _\parallel$ changes from $0\,\%$ to about $-5\,\%$ due to the tension between the data and predictions using lattice $B\rightarrow K^*$ form factors for the branching ratio $\mathcal{B}$ (see also the $\mathcal{B}$ predictions in [49]). We find also substantial negative subleading contributions to the $B\rightarrow K\ell ^+\ell ^-$ amplitude at low $q^2$, which lowers $\langle \mathcal {B}\rangle _{[1,6]}$ to accommodate the measurement by LHCb. This is in agreement with the observations of [36].

Beyond inference of the size of subleading contributions to the amplitudes, we extract information on the $B\rightarrow K$ and $B\rightarrow K^*$ hadronic form factors. We confront our results for the various fit scenarios with the prior values in Table 6. Within the “full” data set, the ratio $V(0)/A_1(0)$ is in overall good agreement with the results labeled “SE2 LCSR” of [31], with less than $1\sigma$ tension for our scenarios SM($\nu$-only) and SM+SM$'$, and $\simeq 1\sigma$ deviation for our scenario SM. Our results for the ratio $A_2(0)/A_1(0)$ are consistently higher than those of [31], with a $2\sigma$ variance which can be attributed to our usage of the constraint on $A_0(0)$; see (6.3). In the case of the “full (+FF)” data set, the prior values of $B\rightarrow K^*$ form factors at $q^2 = 0$ show two to three times smaller uncertainties when taking into account the lattice results [51], whereas the shape parameters $b_1$ are not affected except for the form factor $A_2$. Although lattice results provide a reduction of the prior uncertainty, there is still agreement with [31] within 1$\sigma$ for the “full (+FF)” data set. Except for a slight increase of the variance of $V(0)/A_1(0)$ in scenarios SM and SM+SM$'$, the ratios do not change qualitatively.

**Table 6:** 1D-marginalized posterior results at 68 % probability in comparison to the prior inputs for the various $B\rightarrow K^*$ (upper rows) and $B\rightarrow K$ (middle two rows) form-factor parameters. The results are shown for the “full” (left) and “full (+FF)” (right) data set in various scenarios. The priors for the “full” data set comprise LCSR [35] inputs combined with the additional constraints (6.1)–(6.3) and $B\rightarrow K$ lattice results [50], whereas for “full (+FF)” the $B\rightarrow K^*$ lattice results [51] are added. Note that the marginalization has been performed over all solutions $A,B$ in the case of SM and $A'-D'$ in the case of SM+SM$'$

	No $B\rightarrow K^*$ lattice				$B\rightarrow K^*$ lattice			
	Prior	SM($\nu$-only)	SM	SM+SM$'$	Prior	SM($\nu$-only)	SM	SM+SM$'$
$$V(0)$$	$$ 0.35^{+0.13}_{-0.08}$$	$$ 0.38^{+0.04}_{-0.02}$$	$$ 0.38^{+0.03}_{-0.03}$$	$$ 0.38^{+0.04}_{-0.03}$$	$$ 0.37^{+0.03}_{-0.02}$$	$$ 0.38^{+0.03}_{-0.02}$$	$$ 0.38^{+0.03}_{-0.02}$$	$$ 0.37^{+0.02}_{-0.02}$$
$b_1^V$	$-4.8^{+0.8}_{-0.3}$	$-4.8^{+0.7}_{-0.4}$	$-4.8^{+0.6}_{-0.4}$	$-4.8^{+0.6}_{-0.4}$	$-4.7^{+0.7}_{-0.4}$	$-4.7^{+0.7}_{-0.5}$	$-4.8^{+0.7}_{-0.3}$	$-4.8^{+0.6}_{-0.3}$
$$A_1(0)$$	$$ 0.27^{+0.09}_{-0.05}$$	$$ 0.24^{+0.03}_{-0.02}$$	$$ 0.24^{+0.03}_{-0.03}$$	$$ 0.28^{+0.04}_{-0.03}$$	$$ 0.29^{+0.03}_{-0.03}$$	$$ 0.26^{+0.02}_{-0.02}$$	$$ 0.26^{+0.03}_{-0.02}$$	$$ 0.28^{+0.03}_{-0.03}$$
$$b_1^{A_1}$$	$$ 0.4^{+0.8}_{-0.8}$$	$$ 0.5^{+0.6}_{-0.7}$$	$$ 0.5^{+0.6}_{-0.6}$$	$$ 0.0^{+0.7}_{-0.7}$$	$$ 0.4^{+0.6}_{-0.5}$$	$$ 0.1^{+0.4}_{-0.6}$$	$$ 0.1^{+0.5}_{-0.5}$$	$$ 0.3^{+0.4}_{-0.6}$$
$$A_2(0)$$	$$ 0.24^{+0.13}_{-0.07}$$	$$ 0.23^{+0.04}_{-0.04}$$	$$ 0.22^{+0.05}_{-0.04}$$	$$ 0.27^{+0.06}_{-0.05}$$	$$ 0.29^{+0.05}_{-0.05}$$	$$ 0.27^{+0.03}_{-0.04}$$	$$ 0.26^{+0.04}_{-0.03}$$	$$ 0.28^{+0.04}_{-0.03}$$
$b_1^{A_2}$	$-0.7^{+2.3}_{-1.4}$	$-0.9^{+1.7}_{-1.0}$	$-0.9^{+1.7}_{-1.1}$	$-0.7^{+1.8}_{-1.2}$	$-1.6^{+1.1}_{-0.7}$	$-2.0^{+0.9}_{-0.6}$	$-1.9^{+0.8}_{-0.7}$	$-1.4^{+1.0}_{-0.8}$
$$f_+(0)$$	$$ 0.34^{+0.02}_{-0.02}$$	$$ 0.31^{+0.02}_{-0.01}$$	$$ 0.30^{+0.03}_{-0.01}$$	$$ 0.34^{+0.02}_{-0.02}$$	$$ 0.34^{+0.02}_{-0.02}$$	$$ 0.32^{+0.01}_{-0.02}$$	$$ 0.32^{+0.02}_{-0.02}$$	$$ 0.33^{+0.03}_{-0.02}$$
$b_1^{f_+}$	$-1.7^{+0.4}_{-0.5}$	$-2.3 ^{+0.3}_{-0.3}$	$-2.4^{+0.4}_{-0.4}$	$-1.7^{+0.4}_{-0.5}$	$-1.7^{+0.4}_{-0.5}$	$-2.2^{+0.3}_{-0.4}$	$-2.1^{+0.2}_{-0.4}$	$-1.8^{+0.4}_{-0.4}$
$$V(0)/A_1(0)$$	$$1.25^{+0.41}_{-0.23}$$	$$ 1.31^{+0.31}_{-0.31}$$	$$ 1.57^{+0.20}_{-0.20}$$	$$ 1.29^{+0.21}_{-0.17}$$	$$1.27^{+0.18}_{-0.13}$$	$$ 1.49^{+0.13}_{-0.16}$$	$$ 1.42^{+0.16}_{-0.13}$$	$$ 1.32^{+0.10}_{-0.10}$$
$$A_2(0)/A_1(0)$$	$$0.97^{+0.12}_{-0.12}$$	$$ 0.97^{+0.10}_{-0.15}$$	$$ 0.95^{+0.09}_{-0.07}$$	$$ 0.96^{+0.09}_{-0.06}$$	$$1.00^{+0.08}_{-0.09}$$	$$ 1.02^{+0.06}_{-0.07}$$	$$ 1.00^{+0.07}_{-0.05}$$	$$ 0.99^{+0.05}_{-0.05}$$

### Fit in the SM basis

Although the SM($\nu$-only) fit shows that the SM provides a reasonable description of the available data, we still extend our analysis to obtain model-independent constraints on NP couplings. In the SM scenario we fit the real-valued Wilson coefficients $\mathcal {C}_{7,9,10}^{\mathrm {}}$ in addition to the nuisance parameters (see (2.3)) to the data sets “full”, “full (+FF)” and “selection”. For all data sets we obtain two dominant solutions $A$ and $B$ with SM-like and flipped signs of the Wilson coefficients, and many more solutions with negligible posterior mass.

**Fig. 2:**
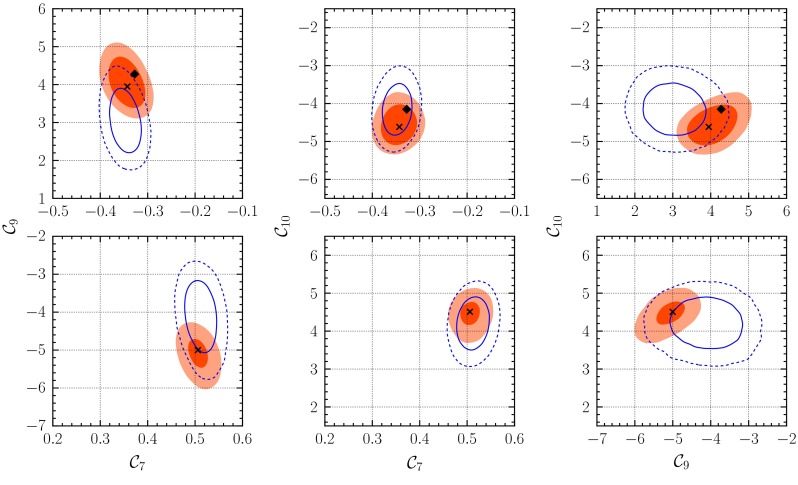
Credibility regions of the Wilson coefficients $\mathcal {C}_{7,9,10}^{\mathrm {}}$ obtained from the fit of the “full” data set after the EPSHEP 2013 conference at $68\,\%$ (*dark red*) and $95\,\%$ (*light red*) probability. The SM-like solution $A$ (*upper row*) and the flipped-sign solution $B$ (*lower row*) are magnified. Overlaid are the results of the fit to the “selection” data set at $68\,\%$ (*blue*, *solid line*) and $95\,\%$ (*blue*, *dashed line*). The *black diamond* and the *black cross* represent the projections of the SM point and the best-fit point to the respective 2D plane

In the case of the “selection” data set, the $p$ values of $0.88$ and $0.89$ are obtained for solutions $A$ and $B$, respectively, depicted in Fig. 2. They are larger compared to $0.75$ obtained in the SM($\nu$-only) scenario, indicating that the additional parameters further reduce the tension with the data by $\Delta \chi ^2 \simeq -5$. Within solution $A$, the fit yields a deviation from the SM value of $\mathcal {C}_{9}^{\mathrm {}}$

4.7 $$\begin{aligned} \Delta _9 = \mathcal {C}_{9}^{\mathrm {}} - \mathcal {C}_{9}^{\mathrm {SM}} \simeq -1.3^{+0.6}_{-0.5}, \end{aligned}$$

with $68\,\%$ probability; see Table 4. However, we find no significant deviations in either $\mathcal {C}_{7}^{\mathrm {}}$ or $\mathcal {C}_{10}^{\mathrm {}}$. This observation is compatible with the findings of [47], where the sign-flipped solution $B$ had been discarded. However, in our results the 2D-marginalized posterior shows merely a $\simeq 2\sigma$ deviation from the SM, in contrast to $3.2\sigma$ as in [47]. The ratio of posterior masses is $R_A : R_B = 47\,\%\,:\,53\,\%$, slightly in favor of the flipped-sign solution.

As in the case of SM($\nu$-only), the $p$ values of the “full” data set are much smaller compared to the “selection” data, but they still indicate a decent fit at $0.08$ for both solutions $A$ and $B$. Contrary to the “selection”, solution $A$ is now strongly favored over solution $B$: $R_A : R_B = 74\,\% : 26\,\%$. This underlines the importance of a combined analysis of all available experimental data rather than a selected subset.

As can be seen in Fig. 2, the SM lies within the $1\sigma$ credibility regions of all 2D-marginalized posterior distributions. With the updated experimental data, the credibility regions are reduced in size by roughly a factor of two when compared to our previous results [30]. For the 1D credibility regions with both solutions $A$ and $B$ we refer to Table 4, whereas for the single solution $A$ we find smaller 68 % probability regions of

$$\begin{aligned} \Delta _7&= 0.0 \pm {0.02},&\Delta _9&= -0.3 \pm {0.4},\\ \Delta _{10}&= -0.4 \pm 0.3. \end{aligned}$$

The authors of [48] do not consider a scenario of simultaneous NP contributions to $\mathcal {C}_{7,9,10}^{\mathrm {}}$, but only single-Wilson-coefficient scenarios $\mathcal {C}_{7}^{\mathrm {}}$ and $\mathcal {C}_{9}^{\mathrm {}}$, the two-Wilson-coefficient scenario $\mathcal {C}_{7,9}^{\mathrm {}}$ and the full set of Wilson coefficients of SM+SM$'$. Their results show a decrease of $|\Delta _7|$ once allowing NP contributions to $\mathcal {C}_{9,10}^{\mathrm {}}$ in the ballpark of our findings.^2^ The NP contributions $\Delta _9$ and $\Delta _{10}$ are also found to be preferentially negative.

The situation of the $P'_5$ anomaly is the same as in the SM($\nu$-only) fit, and the modifications to the posterior distributions of $\zeta ^{L(R) \chi }_{K^*}$, $\chi =\perp ,\parallel ,0$ are of the same type and similar size for both data sets. The same applies to the postdiction $\langle P_5'\rangle _{[1,6]}$ given in Table 2. The pull value of $\langle P'_5\rangle _{[1,6]}$ decreases only little from $2.1\sigma$ in the SM($\nu$-only) fit to $1.6\sigma$ in the SM fit when allowing NP contributions to $\mathcal {C}_{7,9,10}^{\mathrm {}}$. However, the tensions in other measurements are not eased; see Table 5.

Focusing on solution $A$ [cf. (4.5)], the fit yields a Bayes factor of

4.8 $$\begin{aligned} \frac{P(\text {full} | \text {SM})}{P(\text {full} | \text {SM}(\nu -\mathrm{only}))} \Big |_{A} = 1:93. \end{aligned}$$

In the absence of substantial improvements in the handling of subleading contributions to the $B\rightarrow K^{(*)}\ell ^+\ell ^-$ amplitudes, we are forced to conclude that the SM interpretation of the data is more economical than the hypothesis of new physics contributions to the SM Wilson coefficients.

The inclusion of lattice $B\rightarrow K^*$ form factors in the “full (+FF)” analysis shifts the ratio of the probability mass of solutions $A$ and $B$ toward $B$, $R_A :R_B = 0.70 : 0.30$. The $p$ values at the best-fit points decrease to $0.03$ and $0.02$, respectively. Omitting $F_L$ from BaBar and ATLAS, the $p$ values jump to $0.25$ in $A$ and $B$. The 1D-marginalized 68 % probability regions of the Wilson coefficients in solution $A$ remain the same

$$\begin{aligned} \Delta _7&= 0.0\pm 0.02,&\Delta _9&= -0.4^{+0.4}_{-0.3},&\Delta _{10}&= -0.1\pm 0.3, \end{aligned}$$

when compared to the “full” data set, except for the central value of $\Delta _{10}$, which moves even closer to the SM. Finally, the Bayes factor of solution $A$ would be almost unchanged $1:97$.

### Fit in the extended SM+SM$'$ basis

**Fig. 3:**
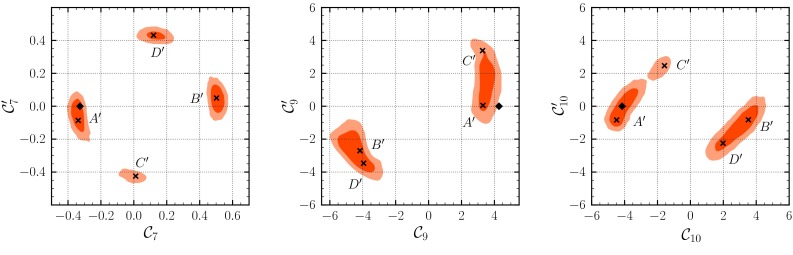
Credibility regions obtained from the fit in the SM+SM$'$ model. We show the results of the full data set after the EPSHEP 2013 conference at $68\,\%$ (*dark red*) and $95\,\%$ (*light red*) probability. The *black diamond* and the *black cross* represent the projections of the SM point and the best-fit point to the respective 2D plane

We proceed with fitting the SM-like and chirality-flipped Wilson coefficients in the SM+SM$'$ scenario. Using the “full” data set we obtain a good fit with $p$ values between $0.07$ and $0.09$ in four well separated solutions $A'$ through $D'$, best seen in the 2D-marginalized $(\mathcal {C}_{7}^{\mathrm {}}-{\mathcal {C}_{7'}^{\mathrm {}}})$ plane in Fig. 3. Here $A'$ and $B'$ denote solutions that show the same signs of the Wilson coefficients $\mathcal {C}_{7,9,10}^{\mathrm {}}$ of the SM operator basis as the solutions $A$ and $B$ in the previous section, and $C'$ and $D'$ denote further solutions. The corresponding $p$ values of the “full (+FF)” data set hardly differ, ranging from $0.06$ to $0.09$. Of all four solutions, $A'$ and $B'$ dominate over $C'$ and $D'$ in terms of the posterior mass:

$$\begin{aligned} R_{A'} : R_{B'} : R_{C'} : R_{D'} = 39\,\% : 41\,\% : 5\,\% : 15\,\%. \end{aligned}$$

The influence of the lattice results of the $B\rightarrow K^*$ form factors roughly increases the posterior mass of solution $A'$ by $10$ % at the expense of $B'$, while leaving $C'$ and $D'$ as is; see Table 3.

The posterior distributions marginalized to the 2D $(\mathcal {C}_{i}^{\mathrm {}} - \mathcal {C}_{i'}^{\mathrm {}})$ planes ($i=7,9,10$) are shown in Fig. 3 with the SM point and the projection of the best-fit points in each solution $A'$ through $D'$. Note that the projection of the best-fit point can deviate from the position of the modes of the marginalized distributions; compare to the 1D intervals in Table 4. Unlike in the SM scenario, it is not possible to disentangle the individual solutions $A'$ through $D'$ within the 1D-marginalized posterior distributions. In order to compare our findings with [48] we choose those intervals that contain the SM-like signs for $\mathcal {C}_{7,9,10}^{\mathrm {}}$ and find with 68 % probability

$$\begin{aligned} \Delta _{7} = +0.01^{+0.02}_{-0.05}, \Delta _{9} = -0.8^{+0.2}_{-0.5}, \Delta _{10} = -0.1^{+0.6}_{-0.5}, \end{aligned}$$

in agreement with [48]. Our results hardly change when taking $B\rightarrow K^*$ lattice results into account. The best-fit points for $\mathcal {C}_{7',9',10'}^{\mathrm {}}$ of [48] fall into the intervals given in Table 4, with larger deviations from the modes of the 1D posterior distributions. The SM prediction $\mathcal {C}_{7'}^{\mathrm {SM}} = -0.01$ and $\mathcal {C}_{10'}^{\mathrm {SM}} = 0$ is contained in the smallest 68 % region of the 1D-marginalized posterior distributions, whereas $\mathcal {C}_{9'}^{\mathrm {SM}} = 0$ is not. In the 2D-marginalized ($\mathcal {C}_{9}^{\mathrm {}}$—$\mathcal {C}_{9'}^{\mathrm {}}$) plane, the SM point is just outside of the 95 % region, dominantly due to a shift in $\mathcal {C}_{9}^{\mathrm {}}$; see Fig. 3.

The additional NP contributions in chirality-flipped operators in scenario SM+SM$'$ can address the tension in the measurement of $\langle P_5' \rangle _{[1,6]}$. However, the previously mentioned large pull values for $\langle F_L \rangle _{[1,6]}$, $\langle \mathcal{B}\rangle _{[16,19]}$, $\langle P_4' \rangle _{[14.18,16]}$ and $\langle A_\mathrm{FB} \rangle _{[16,19]}$ remain almost unchanged (see Table 5). This corroborates the findings of [31, 48] that the pull value of $\langle P_4'\rangle _{[14.18,16]}$ cannot be pushed below $2\sigma$.

Focusing on the solution $A'$ [cf. (4.5)], the Bayes factor becomes

4.9 $$\begin{aligned} \frac{P(\text {full} | \text {SM+SM'})}{P(\text {full} |\text {SM}(\nu -\mathrm{only}))}\Big |_{A'} = 1:19. \end{aligned}$$

Thus the NP hypothesis with chirality-flipped Wilson coefficients is disfavored in comparison to the SM($\nu$-only) hypothesis. However, the data favor SM+SM$'$ over SM with roughly $5:1$. Taking the lattice form factor results into account, we find

4.10 $$\begin{aligned} \frac{P(\text {full (+FF)} | \text {SM+SM'})}{P(\text {full (+FF)} | \text {SM}(\nu -\mathrm{only}))}\Big |_{A'} = 5:1, \end{aligned}$$

a significant increase by almost 100 compared to (4.9); see the discussion in Sect. 4.5.

In the SM+SM$'$, the size of subleading contributions to transversity amplitudes $\chi = 0\, (\parallel )$ reduces to about $-5\,\%$ ($+5\,\%$) for $\zeta _{K^*}^{L \chi }$, in contrast to $\zeta _{K^*}^{L \perp }$, which remains large; see Fig. 1. The solutions $A'$ through $D'$ are clearly distinct when viewed in the $\zeta _{K^*}^{L \perp } - \mathcal {C}_{9'}^{\mathrm {}}$ plane. The subleading parameter $\Lambda _\parallel$ at high $q^2$ decreases back to 0 % for the “full (+FF)” data set. There are no differences between the “full” and “full (+FF)” data sets. Similarly, the subleading contributions to the $B\rightarrow K\ell ^+\ell ^-$ amplitude disappear. The small shifts we observe between the SM and SM+SM$'$ scenarios suggest a common property to ease the tensions between predictions and data, that is shared by the $\zeta ^{L\chi }_{K^*}$ and the chirality flipped Wilson coefficients. It is therefore desirable to better understand size, chirality structure, and $q^2$-dependence of the power corrections.

**Fig. 4:**
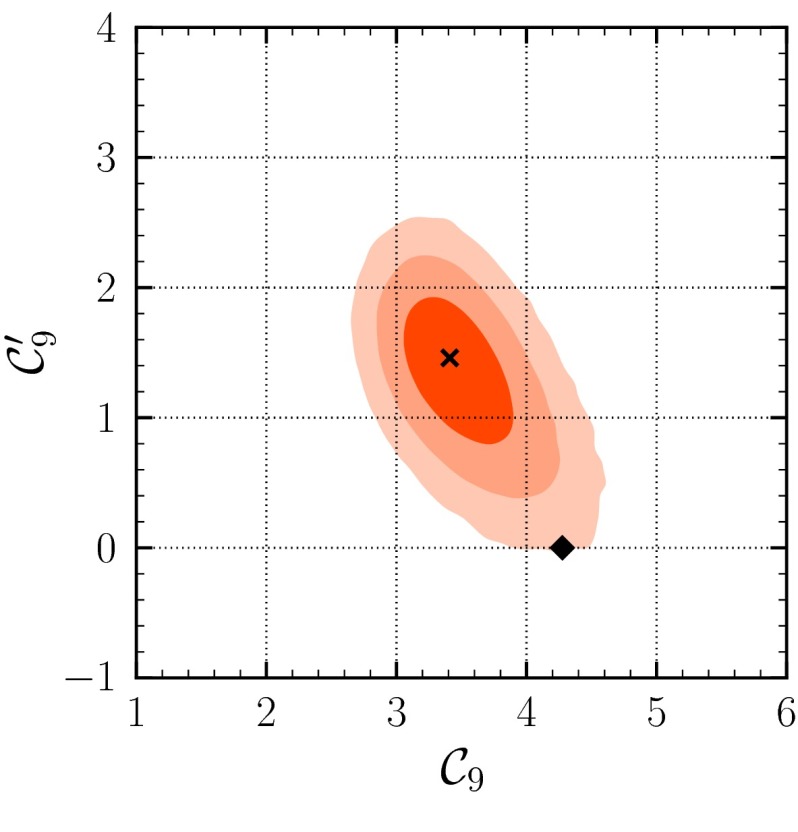
Credibility regions obtained from the fit in the SM+SM$'(9)$ model. We show the results of the full data set after the EPSHEP 2013 conference at $68.3\,\%$ (*dark red*), $95.4\,\%$ (*light red*), and $99.7\,\%$ (*lightest red*) probability. The *black diamond* and the *black cross* represent the projections of the SM point and the best-fit point

In view of the significantly enlarged Bayes factor (4.9) in the scenario with chirality-flipped operators compared to (4.8) in the SM, we investigate also the variant SM+SM$'(9)$. This scenario has only two additional new-physics parameters $\mathcal {C}_{9,9'}^{\mathrm {}}$ compared to six in the SM+SM$'$ scenario, implying a “smaller punishment” due to the larger prior volume w.r.t. the SM($\nu$-only) case. The 2D-marginalized $(\mathcal {C}_{9}^{\mathrm {}}-\mathcal {C}_{9'}^{\mathrm {}})$ plane in Fig. 4 shows the SM point on the border of the $3\sigma$ region for the only solution $A'$. The results do not change much when using the “full (+FF)” data set. The $1\sigma$ probability regions from the 1D-marginalized posterior distributions are

$$\begin{aligned} \Delta _{9}&= -0.8 \pm 0.3, \qquad \Delta _{9'} = +1.4^{+0.3}_{-0.4}, \end{aligned}$$

which is compatible with the results of [48]. With the same parameter ranges as in (4.9), the Bayes factor with the “full” data set comparing the SM-like solution $A'$ to SM($\nu$-only) is

4.11 $$\begin{aligned} \frac{P(\text {full} | \text {SM+SM'(9)})}{P(\text {full} | \text {SM}(\nu -\mathrm{only}))}\Big |_{A'} = 8:1, \end{aligned}$$

slightly favoring the SM+SM$'(9)$ over the SM. With the “full (+FF)” data set, the Bayes factor increases to

4.12 $$\begin{aligned} \frac{P(\text {full (+FF)} | \text {SM+SM'(9)})}{P(\text {full (+FF)} | \text {SM}(\nu -\mathrm{only}))}\Big |_{A'} = 820:1. \end{aligned}$$

### Interpretation

We conclude this section with a remark on the model comparison. We emphasize again that, to derive the posterior odds, the Bayes factor has to be multiplied by the appropriate prior odds [see (4.4)], the determination of which is beyond the scope of the present work. Given that the standard model successfully describes the vast majority of particle physics data, our prior odds are in strong favor of the SM($\nu$-only). This is true even for the considered rare decays despite their particular sensitivity to new physics because the majority of standard model predictions are in the ballpark of the other experimental measurements of $\Delta B = 1, 2$ observables.

Currently the lack of experimental evidence for right-handed weak interactions significantly reduces our prior probabilities of scenarios with chirality-flipped Wilson coefficients SM+SM$'$ and SM+SM$'(9)$ compared to SM($\nu$-only). Among the scenarios with chirality-flipped Wilson coefficients we would set prior odds in favor of SM+SM$'$ because SM+SM$'(9)$ is only a restricted subset of SM+SM$'$.

It remains to be noted that the SM+SM$'(9)$ scenario can be realized in a model-independent way due to operator mixing with $b\rightarrow s f\bar{f}$ four fermion operators. Such scenarios have been considered previously for $f = \text{ quarks }$ [64–66] as well as $f =\tau$ [67] and recently discussed in the present context of the data [68] for $f = b$. In the case of $f= \text{ quarks }$ also hadronic charmless decays would be affected [69]. Explicit $Z'$-models have been discussed recently in [70–73].

We find for nearly all combinations of scenarios and data sets, except the “selection” data set, satisfactory $p$ values of about 5–$10\,\%$. This indicates that it is possible to find a single parameter point (the best-fit point), at which the data is adequately described. The Bayes factor, in contrast, quantifies the ability to describe the data, averaged over the full parameter space. Concerning the SM scenario, the Bayes factor indicates that the additional parameters do not yield an advantage over the SM($\nu$-only) scenario. However, it is interesting to see that the Bayes factors are undecided or even in favor of the scenarios SM+SM$'$ and SM+SM$'(9)$ for the “full” data set. Taking lattice results into account, the Bayes factors increase roughly by a factor $100$ for the “full (+FF)” data set.

We emphasize that this increase in the latter scenarios for the “full (+FF)” data set can be traced to three causes. First, the prior information based on the lattice results is in line with the information on form factor parameters as inferred from the data; see Table 6. Second, chirality-flipped Wilson coefficients can, at least partially, take on the role of subleading contributions to the $B\rightarrow K^{(*)}\ell ^+\ell ^-$ amplitudes. Since changing the Wilson coefficients (with flat priors) is “less costly” than changing the subleading parameters (with Gaussian prior distributions), the Bayes factor penalizes the latter. Third, the goodness of fit decreases for SM($\nu$-only) while it stays constant for SM+SM$'$ as follows from the $\chi ^2$ values of Table 3.

Evaluating the sensitivity to the prior shape, we repeated the “full (+FF)” fits replacing Gaussian priors with flat priors of the same range—as in Fig. 1—for all subleading parameters. The Wilson coefficients are determined with the same or slightly higher precision as before. Most of the subleading parameters play a minor role in the fit, so the prior equals the posterior. The two exceptions are the large-recoil parameters $\zeta ^{L\perp }_{K^*}$ and $\zeta ^{L0}_{K^*}$. Their posteriors are very similar to those of the fit with Gaussian priors shown in Fig. 1 but marginally wider. Due to the extra freedom, all models are able to better describe the data. At the best-fit point, the $p$ values roughly double from 0.04 to 0.1 (SM($\nu$-only)), from 0.09 to 0.16 (SM+SM$'$), and from 0.12 to 0.2 (SM+SM$'(9)$). The Bayes factors slightly shift in favor of SM($\nu$-only) by 2.6 (SM+SM$'(9)$) and 4 (SM+SM$'$) indicating that the models with variable $\mathcal {C}_{i}^{\mathrm {}}$ on average do not benefit as much from the added flexibility. In summary, changing the prior shape of the subleading parameters does not entail any big surprise and corroborates our main findings.

## Conclusions

Our Bayesian analysis indicates that the standard model provides an adequate description of the available measurements of rare leptonic, semileptonic, and radiative $B$ decays. Compared to our previous analysis [30], we determine the Wilson coefficients $\mathcal {C}_{7,9,10}^{\mathrm {}}$ more accurately, dominantly due to the reduction of the experimental uncertainties in the exclusive decays and the addition of the inclusive decay $B\rightarrow X_s\gamma$.

Contrary to all similar analyses, our fits include the theory uncertainties explicitly through nuisance parameters. We observe that tensions in the angular and optimized observables in $B\rightarrow K^*\ell ^+\ell ^-$ decays can be lifted through $(10$–$20)\,\%$ shifts in the transversity amplitudes at large recoil due to subleading contributions. These shifts are present within the SM as well as the model-independent extension of real-valued Wilson coefficients $\mathcal {C}_{7,9,10}^{\mathrm {}}$. For the scenarios introducing additional chirality-flipped coefficients $\mathcal {C}_{7',9',10'}^{\mathrm {}}$, the shifts reduce to a few percent (except $\zeta _{K^*}^{L\perp }$). We find $|\mathcal {C}_{9'}^{\mathrm {}}| < 4\,(4.1)$ and $|\mathcal {C}_{10'}^{\mathrm {}}| < 2\, (3)$ with $68\,\%$ ($95\,\%$) probability for the right-handed couplings, which holds in the absence of scalar and tensor contributions. These constraints are insensitive to the shape (Gaussian vs. flat) of the priors of subleading corrections.

Among the information inferred from the data are constraints on the parameters of the $B\rightarrow K^{(*)}$ form factors. We have performed all fits with and without the very recent lattice $B\rightarrow K^*$ form factor predictions [51]. These form factors constitute very strong additional prior information leading to a tension with branching fraction measurements of $B\rightarrow K^*\ell ^+\ell ^-$ at high $q^2$ [49]. It can be eased by the presence of chirality-flipped Wilson coefficients and currently disfavor new physics without them. Notably, in the fits without the lattice results the data still yields larger posterior form factor values in scenarios with chirality-flipped Wilson coefficients, such that independently of the inclusion of the lattice predictions, the posterior ranges of the Wilson coefficients differ only little in these scenarios.

The rough picture emerging from current data may be summarized as follows. The low-$q^2$ $B\rightarrow K^*\ell ^+\ell ^-$ data prefer a negative new-physics contribution to $\mathcal {C}_{9}^{\mathrm {}}$ [47], which is not supported by $B\rightarrow K \ell ^+\ell ^-$ data unless one allows a positive contribution to $\mathcal {C}_{9'}^{\mathrm {}}$ (or alternatively $\mathcal {C}_{10'}^{\mathrm {}}$) [48]. The additional $\mathcal {C}_{9'}^{\mathrm {}}$ would be in conflict with current measurements of the branching fraction of $B\rightarrow K^* \ell ^+\ell ^-$ at high $q^2$, unless one increases the $B\rightarrow K^*$ form factors $A_{1,2}$ by about 15 % (see Table 6), which is well within the theoretical uncertainties of their extrapolations of light-cone sum rule predictions from low to high $q^2$. Perhaps not accidentally, the central values of recent lattice predictions for these form factors at high $q^2$ [49] are also higher by the same amount, whereas their theoretical uncertainties are below 10 %. Including the lattice predictions, the model comparison suggests that scenarios with chirality-flipped Wilson coefficients can provide an explanation of the data as efficient or even better than the standard model.

A substantial reduction of uncertainties can be expected for LHCb, CMS, and ATLAS measurements of $B^0\rightarrow K^{*0}\ell ^+\ell ^-$ and $B^+\rightarrow K^+\ell ^+\ell ^-$ once they publish the analysis of their 2012 data sets. It should also be mentioned that $B\rightarrow K^*\gamma$ and $B\rightarrow K^{(*)} \ell ^+\ell ^-$ results from Belle are not based on the final reprocessed data set and that BaBar’s angular analysis of $B\rightarrow K^*\ell ^+\ell ^-$ is still preliminary. It remains to be seen whether these improved analyses further substantiate the present hints of a $2\sigma$ deviation from the SM prediction in the $(\mathcal {C}_{9}^{\mathrm {}}$—$\mathcal {C}_{9'}^{\mathrm {}})$ plane.

In our opinion, however, there remain two major challenges on the theory side. The first is to improve our analytic knowledge of the $1/m_b$ corrections to the exclusive decay amplitudes. The second is to reduce the uncertainty from hadronic form factors, especially at low $q^2$. Without improvements on either, there is little prospect to discern between small NP effects and large subleading corrections. Another point of concern are potentially large duality-violating effects that render the OPE at high $q^2$ invalid. They have been estimated, though model-dependently, to be small [40]. In this regard, the experimental verification of certain relations [28] among angular observables in $B\rightarrow K^*\ell ^+\ell ^-$ that are predicted by the OPE would be very desirable. In the case that some of these relations are not fulfilled, the analysis of the breaking pattern can provide information on duality violation but also on additional new-physics scalar and tensor interactions.

## Appendix A: Theoretical treatment

This appendix details the theoretical predictions for newly added observables, and their respective nuisance parameters are discussed. Further we explain changes to the choice of priors for some of the nuisance parameters, as well as a different parametrization of $B\rightarrow K^*$ form factors, always with respect to our previous analysis [30]. We also list the updated values of input parameters whose uncertainty is neglected in Table 7.

**Table 7:** Numerical input that has been updated but is not used as nuisance parameters in the fit. $^\dagger$See text for additional details

Quantity	Unit	Value	Reference
$\alpha _s(M_Z)$	$0.1184$	–	[54]
$M_Z$	$91.1876$	GeV	[54]
$M_W$	$80.385$	GeV	[54]
$m_t^\mathrm{pole}$	$173.5$	GeV	[54]
$M_{B^{+}}$	$5.27925$	GeV	[54]
$M_{B^{0}}$	$5.27958$	GeV	[54]
$\tau _{B^{+}}$	$1.641$	ps	[54]
$\tau _{B^{0}}$	$1.519$	ps	[54]
$\tau _{B^{+/0}}$	$1.580$	ps	$^\dagger$
$f_{B^{+/0}}$	$190.6 \pm 4.7$	MeV	[75]
$M_{B_s}$	$5.36677$	GeV	[54]
$\tau _{B_s}$	$1.516$	ps	[54, 76]
$\Delta \Gamma _s$	$0.081$	ps$^{-1}$	[54, 76]
$y_s$	$0.062$	–	[54, 76]

Concerning the set of common nuisance parameters—the Wolfenstein parameters of the CKM-quark-mixing matrix and the $\overline{\mathrm{MS}}$ bottom- and charm-quark masses entering the majority of observables—we use the updated values given in Table 8 based on the most recent PDG combinations [54] and the tree-level result of the UTfit collaboration [74].

**Table 8:** Prior distributions of common nuisance parameters

Quantity	Prior	Unit	Reference
CKM			
$\lambda$	$0.22535 \pm 0.00065$	–	[74]
$A$	$0.807 \pm 0.020$	–	[74]
$\bar{\rho }$	$0.128 \pm 0.055$	–	[74]
$\bar{\eta }$	$0.375 \pm 0.060$	–	[74]
Quark masses			
$\overline{m}_c(m_c)$	$1.275 \pm 0.025$	$\mathrm {GeV}$	[54]
$\overline{m}_b(m_b)$	$4.18 \pm 0.03$	$\mathrm {GeV}$	[54]

We model asymmetric uncertainty intervals for the priors with LogGamma distributions (see [30] for details), while symmetric intervals are implemented as Gaussian priors.

### Appendix A.1: Inclusive decays $B\rightarrow X_s (\gamma ,\, \ell ^+\ell ^-)$

The branching ratio of the inclusive decay $B\rightarrow X_s \gamma$, $\mathcal {B}(B\rightarrow X_s \gamma ) \sim |\mathcal {C}_{7}^{\mathrm {}}|^2 + |\mathcal {C}_{7'}^{\mathrm {}}|^2$, represents the most stringent constraint on the magnitude of the dipole Wilson coefficients $\mathcal {C}_{7,7'}^{\mathrm {}}$. In our analysis we include the known corrections to next-to-leading order in $\alpha _s$ [77, 78] as well as $\alpha _s \Lambda _\mathrm{QCD}^2/m_b^2$ corrections [79]. Contrary to the common normalization to the semileptonic inclusive decay $B\rightarrow X_c \ell \nu$, we express the branching ratio in terms of the averaged $B$ meson life time for a 50:50 production ratio of $B^+B^-$ to $B^0 \bar{B}^0$ pairs at the $\Upsilon (4S)$, $\tau _{B^{+/0}}$ given in Table 7, and the bottom-quark pole mass. In order to avoid renormalon ambiguities we calculate the pole mass value from the $\overline{\mathrm{MS}}$ mass $m_b(m_b)$ using the 3-loop result [80]. The $\overline{\mathrm{MS}}$ mass is part of our set of common nuisance parameters and its uncertainty dominates the overall theory uncertainty of inclusive decays. At order $\Lambda _\mathrm{QCD}^2/m_b^2$ in the heavy-quark expansion hadronic matrix elements of two dimension-five operators enter. They are parametrized in terms of $\mu ^2_{\pi }$ and $\mu ^2_{G}$ for the expectation values of the kinetic and the chromomagnetic operators, respectively. The parameters $\mu ^2_\pi$ and $\mu ^2_G$ enter the fit with priors according to [81], which are listed in Table 9. The correlation between $\mu ^2_{G}$ and the $b$-quark mass is accounted for by the $B^*$–$B$ mass splitting. When confronting the theoretical prediction of the mass splitting with the measurement (3.5), we include also the effect of dimension-six operators as described in [81]. Our SM prediction $\mathcal {B}(B\rightarrow X_s \gamma ) = (3.14^{+0.22}_{-0.19})\cdot 10^{-4}$ is in good agreement with the NNLO result [82]. The theory uncertainty of our prediction is determined from variation of the Wolfenstein parameters, $m_b(m_b)$, $m_c(m_c)$, $\mu ^2_{\pi }$, and $\mu ^2_{G}$.

**Table 9:** Prior distributions of the nuisance parameters for hadronic quantities entering inclusive and exclusive decays

Quantity	Prior	Unit	Reference
Inclusive decays			
$\mu ^2_{\pi }(1\,\text{ GeV })$	$0.45 \pm 0.10$	$\mathrm {GeV}^2$	[81]
$\mu ^2_{G}(1\,\text{ GeV })$	$0.35^{+0.03}_{-0.02}$	$\mathrm {GeV}^2$	[81]
$B\rightarrow K$ form factors			
$f_+(0)$	$0.34 \pm 0.05$	–	[35, 84]
$b_1^+$	$-2.1^{+0.9}_{-1.6}$	–	[35]
$B\rightarrow K^{*}$ form factors			
$V(0)$	$0.36^{+0.23}_{-0.12}$	–	[35]
$A_1(0)$	$0.25^{+0.16}_{-0.10}$	–	[35]
$A_2(0)$	$0.23^{+0.19}_{-0.10}$	–	[35]
$b_1^V$	$-4.8^{+0.8}_{-0.4}$	–	[35]
$b_1^{A_1}$	$0.34^{+0.86}_{-0.80}$	–	[35]
$b_1^{A_2}$	$-0.85^{+2.88}_{-1.35}$	–	[35]
$B_s$ decay constant			
$f_{B_s}$	$227.6 \pm 5.0$	MeV	[75, 85–87]

For the prediction of the branching ratio of the inclusive decay $B\rightarrow X_s \ell ^+\ell ^-$ we work at NNLO in QCD and NLO in QED, including also $\Lambda _\mathrm{QCD}^2/m_b^2$ subleading corrections, as described in [52, 53] except for the SM-SM$'$ interference terms. We adopt the same normalization in terms of $\tau _{B^{+/0}}$ and bottom-quark pole mass as described for $B\rightarrow X_s \gamma$. The chirality-flipped operators are included following [83] and NLO QCD corrections to matrix elements are accounted for in case they can be derived easily within the SM operator basis. The overall theory uncertainty is determined as for $B\rightarrow X_s\gamma$ from the variation of the same nuisance parameters. We obtain as the SM prediction $\langle \mathcal {B}(B\rightarrow X_s \ell ^+ \ell ^-)\rangle _{[1,\,6]} = (1.4\pm 0.1) \cdot 10^{-6}$.

### Appendix A.2: Form factors and decay constants

The most important change in the treatment of form factors is the consistent use of the parametrization as in [35], for both $B\rightarrow K$ and $B\rightarrow K^*$ transitions. It has the merits of (a) a convenient expansion in a small parameter $z$ that respects unitarity, (b) correct behavior at the $BK^{(*)}$ production threshold, (c) correct asymptotic behavior for $q^2\rightarrow \infty$, and (d) a convenient parametrization at $q^2 = 0$.

For $B\rightarrow K$ we have modified the prior of the nuisance parameter $f_+(0)$ of the $f_+$ form factor parametrization w.r.t. our previous analysis but kept the slope parameter $b_1^+$ as is. This change accounts for both LCSR results [35, 84] that use the same approach of $B$-interpolating currents and on-shell $K$-mesons. As a result the prior on $f_+(0)$ is wider and the tension between the SM prediction of the $B\rightarrow K \ell ^+\ell ^-$ branching ratio at $q^2 \in [1, 6]$ GeV$^2$ [36] and the LHCb measurement [15] is reduced. Moreover, the recent lattice predictions [50] of the form factor $f_+$ at high $q^2$ are included in our analysis as part of the likelihood for technical reasons. For this purpose we reproduced lattice predictions at three values of $q^2 = 17,\, 20,\, 23$ GeV$^2$ as well as their correlation matrix based on the parametrization given in [50]; see Table 10. (The $q^2$ values and number of points are chosen such that the correlation of neighboring points does not exceed $80\,\%$.) This constraint is included in the likelihood by means of a multivariate Gaussian.

**Table 10:** Reproduction of mean values, uncertainties (*top*) and correlation information (*bottom*) of lattice points [50] for the vector form factor $f_+(q^2)$ in $B\rightarrow K$ transitions

$q^2$ ($\mathrm {GeV}$)	$17$	$20$	$23$
$f_+(q^2)$	$1.08 \pm 0.03$	$1.51 \pm 0.03$	$2.34 \pm 0.07$
$17$	$1.00$	$0.78$	$0.29$
$20$	–	$1.00$	$0.71$
$23$	–	–	$1.00$

Due to the change of parametrization of the $B\rightarrow K^*$ form factors $V, A_1$ and $A_2$, their three respective nuisance parameters are replaced by the three form factor normalizations at $q^2 = 0$ ($V(0), A_{1,2}(0)$) and three slope parameters ($b_1^{V, A_{1,2}}$). The LCSR results of [35] are chosen as the priors for normalizations and slopes. We note that these priors are less precise than the results of [88] due to a novel LCSR setup involving an on-shell $B$ meson and interpolation of the $K^*$ final state. Beyond the informative priors we also include two additional constraints on $B\rightarrow K^*$ form factors at $q^2 = 0$. First, the ratio $V(0)/A_1(0)$ is constrained in the large energy limit as given by [31] (see also references therein)

6.1 $$\begin{aligned} V(0)/A_1(0)&= 1.33\pm 0.40, \end{aligned}$$

where the uncertainty has been estimated based on power counting. Second, we make use of the relation

6.2 $$\begin{aligned} A_0(0) = \frac{M_B + M_{K^*}}{2 M_{K^*}} A_1(0) - \frac{M_B - M_{K^*}}{2 M_{K^*}} A_2(0) \end{aligned}$$

where

6.3 $$\begin{aligned} A_0(0) = 0.29^{+0.10}_{-0.07} \end{aligned}$$

is the LCSR result given in [35]. We note that (6.1) and (6.3) represent additional constraints on the nuisance parameters $V(0)$ and $A_{1,2}(0)$. The motivation for this treatment is to tighten the constraints on $A_{1}(0)$, and to avoid unphysical (i.e. negative) values of the form-factor combination $A_0$.

Similar to our treatment of $B\rightarrow K$ lattice results, we reproduce lattice predictions [51] for the $B\rightarrow K^*$ form factors $V$, $A_1$, and $A_{12}$ at two kinematic points $q^2 = 15,\, 19.21$ GeV${}^2$. We obtain mean values, variances, and the correlation coefficients between neighboring points as given in Table 11. Note that Ref. [51] does not provide information on cross-form factor correlation. We chose the number and spacing of $q^2$ values so that the correlation of neighboring points does not exceed $60\,\%$.

**Table 11:** Reproduction of mean values, uncertainties (*top*) and correlation information (*bottom*) of lattice points based on the results in the arXiv v1 of [51] for the vector form factors $V(q^2)$ and $A_{1,12}(q^2)$ in $B\rightarrow K^*$ transitions. Note that the update of the results from the arXiv v1 to v2 introduce only minor changes to the mean values and the correlation coefficient for the $A_{12}$ lattice points

$q^2$ ($\mathrm {GeV}$)	$15$					$19.21$
$V(q^2)$	$1.19 \pm 0.10$					$1.98 \pm 0.11$
$A_1(q^2)$	$0.52 \pm 0.03$					$0.64 \pm 0.02$
$A_{12}(q^2)$	$0.36 \pm 0.03$					$0.43 \pm 0.03$

	$V$		$A_1$		$A_{12}$	
$q^2$ ($\mathrm {GeV}$)	$15$	$19.21$	$15$	$19.21$	$15$	$19.21$
$15$	$1.00$	$0.46$	$1.00$	$0.52$	$1.00$	$0.25$
$19.21$	–	$1.00$	–	$1.00$	–	$1.00$

The updated prior of the $B_s$ decay constant $f_{B_s}$, entering the branching ratio of $B_s\rightarrow \mu ^+\mu ^-$, takes into account recent lattice results; see Table 9.

### Appendix A.3: Subleading $1/m_b$

With increasing knowledge of the $B\rightarrow K^{(*)}$ form factors and measurement of optimized—i.e., form factor insensitive—observables, the treatment of subleading contributions to the amplitudes of both $B\rightarrow K\ell ^+\ell ^-$ and $B\rightarrow K^*\ell ^+\ell ^-$ decays has increased in relevance. Especially their analytic $q^2$ dependence is currently unknown, and their determination is not within the scope of this work. However, we strive to infer the size of contributions that go beyond the known QCDF and low-recoil terms. In order to achieve this goal we keep the parametrization of subleading terms as in our previous work, except for the complex phases of the low-recoil terms. Inference of these phases is not possible in the absence of data on CP asymmetries in $B\rightarrow K^{(*)}\ell ^+\ell ^-$ decays at low recoil [30]. We therefore remove these phases from the analysis.

The overall theory uncertainty of our predictions in the region of large recoil differ substantially from those given in [38], due to the different treatment of subleading corrections to the form factor relations and the contributions from $c\bar{c}$ resonances. We keep the parametrization as in our previous work,

6.4 $$\begin{aligned} A^{L(R)}_\chi&\mapsto A^{L(R)}_\chi \zeta ^{L(R)\chi }_{K^*},&\chi&=\, \perp ,\parallel , 0, \end{aligned}$$

and obtain similar uncertainties in predictions of observables as in [47].

We collect the experimental measurements as well as theoretical predictions from the literature and this work in Table 2 for the optimized observables $\langle P_{4,5,6}' \rangle$. There we give predictions, i.e., before the fit, and postdictions that include experimental information, and proceed for this purpose as described in [30]. The predictions are restricted to the SM($\nu$-only) scenario and are based on the prior distributions of the nuisance parameters. Besides our nominal Gaussian prior choice with $1\sigma$ ranges of $\pm 0.15$ ($15\,\%$ at amplitude level) for subleading parameters at large recoil, $\zeta ^{L(R)\chi }_{K^*,K}$, and also at low recoil, we also show the results for wider $1\sigma$ range of $\pm 0.45$. We do not include the recent lattice results of $B\rightarrow K^*$ form factors [51].

The central values of our predictions agree within errors with [38, 47, 49]. At large recoil our theoretical uncertainties are of the same size as in [47] and much smaller than in [38], which treats subleading corrections differently. Choosing wider prior ranges leads to small shifts in the central value and can double the theoretical uncertainty that is still smaller than the one in [38]. At low recoil, subleading corrections are less important and the wider prior ranges practically do not affect the overall uncertainties.

The postdictions are based on the posterior distributions of the fits for each scenario with nominal prior distributions. This includes NP effects in the Wilson coefficients in the scenarios SM and SM+SM$'$. The overall uncertainties of the postdictions are smaller than the uncertainties of the predictions. This can be attributed to the improved knowledge of form factors and subleading nuisance parameters as witnessed by the prior-to-posterior compression in Table 6 and Fig. 1. The additional NP contributions in SM compared to SM($\nu$-only) do not change postdictions of $\langle P_{4,5,6}' \rangle$ noticeably. On the other hand, chirality-flipped operators in SM+SM$'$ can induce small shifts only at low recoil.

## Footnotes

1 This does not apply to NP scenarios with additional (pseudo-) scalar and tensor operators, however, the present LHCb analysis [19] cannot be applied to such scenarios.

2 Note that in [48] Wilson coefficients are determined at the scale $\mu = 160$ GeV but RGE effects are only of concern for $\Delta _7$.
